# Supplementary material for: Climate econometric models indicate solar geoengineering would reduce inter-country income inequality
Source: Nat Commun. 2020 Jan 13;11:227. doi: 10.1038/s41467-019-13957-x (PMC6957473; doi:10.1038/s41467-019-13957-x)
Supplement: Supplementary file 2 — Supplementary Information [file 41467_2019_13957_MOESM2_ESM.pdf]

Supplementary Materials for "Climate econometric models indicate solar geoengineering would reduce inter-country income inequality"

Anthony R. Harding[1,2], Katharine Ricke[1,3*], Daniel Heyen[4], Douglas G. MacMartin[5], Juan Moreno-Cruz[6]

[1]School of Global Policy and Strategy, University of California, San Diego
[2]Department of Economics, Georgia Institute of Technology
[3]Scripps Institution of Oceanography, University of California, San Diego
[4]Center of Economic Research, ETH Zürich
[5]Mechanical and Aerospace Engineering, Cornell University
[6]School of Environment, Enterprise and Development, University of Waterloo
*Correspondence to: kricke@ucsd.edu

Supplementary Figures

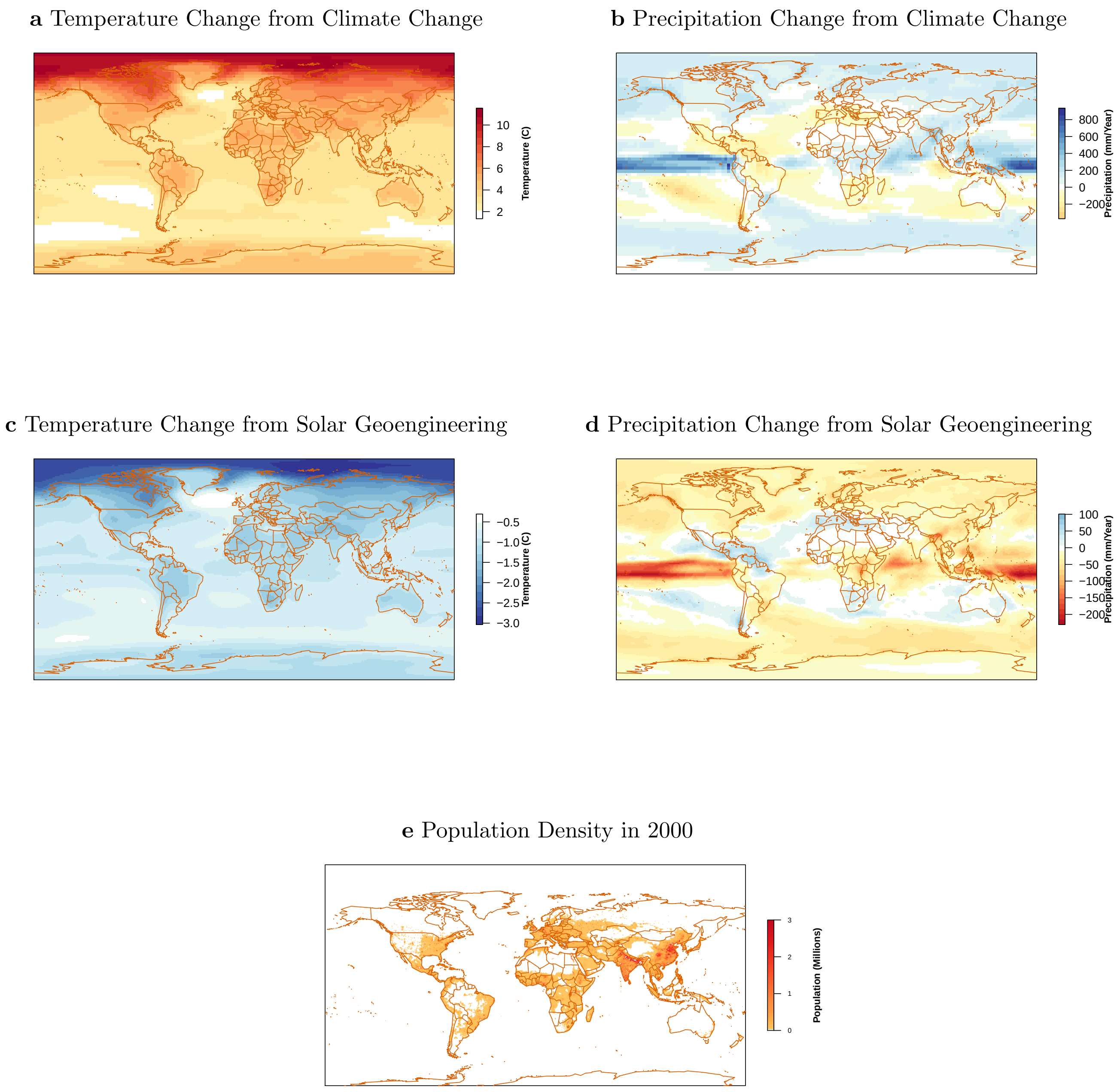

Supplementary Figure 1 | Climate Projections a, ensemble mean of temperature projections for change in near-surface temperature from 2081-2100 relative to 1986-2005 for models participating in CMIP5. **b**, ensemble mean of precipitation projections for change in precipitation from 2081-2100 relative to 1986-2005 for models participating in CMIP5. **c**, ensemble mean of temperature change from a 1°C change in temperature from solar geoengineering for 12 models participating in the GeoMIP G1 experiment. **d**, ensemble mean of precipitation change from a 1°C change in temperature from solar geoenginxeering for 12 models participating in the GeoMIP G1 experiment. **e**, grid-cell polulation density in year 2000 used for calculating population-weighted country mean values of temperature and precipitation.

a.

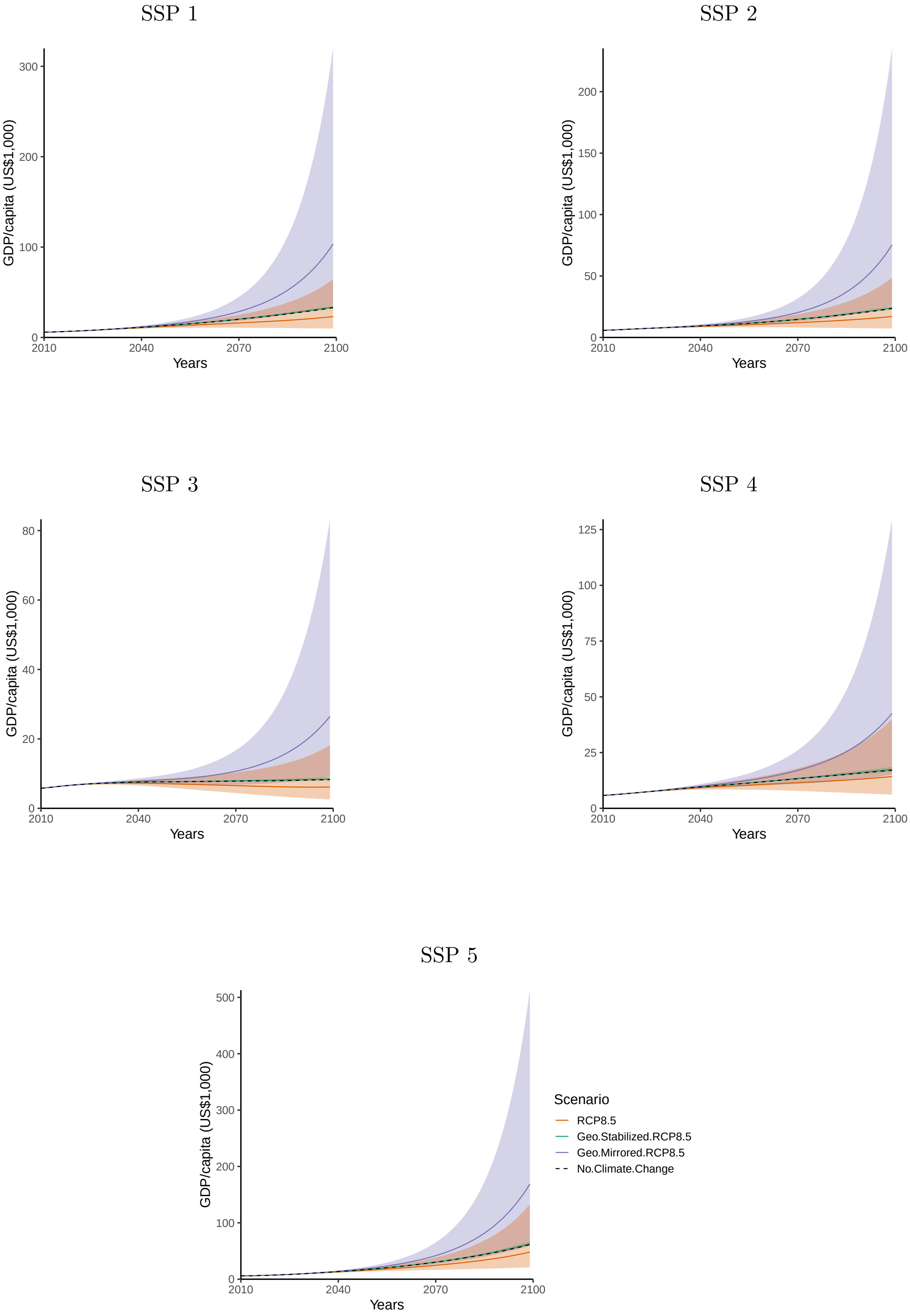
SSP 1
SSP 2
SSP 3
SSP 4
SSP 5
GDP/capita (US$1,000)
Years
2010
2040
2070
2100
Scenario
RCP8.5
Geo.Stabilized.RCP8.5
Geo.Mirrored.RCP8.5
No.Climate.Change

b.

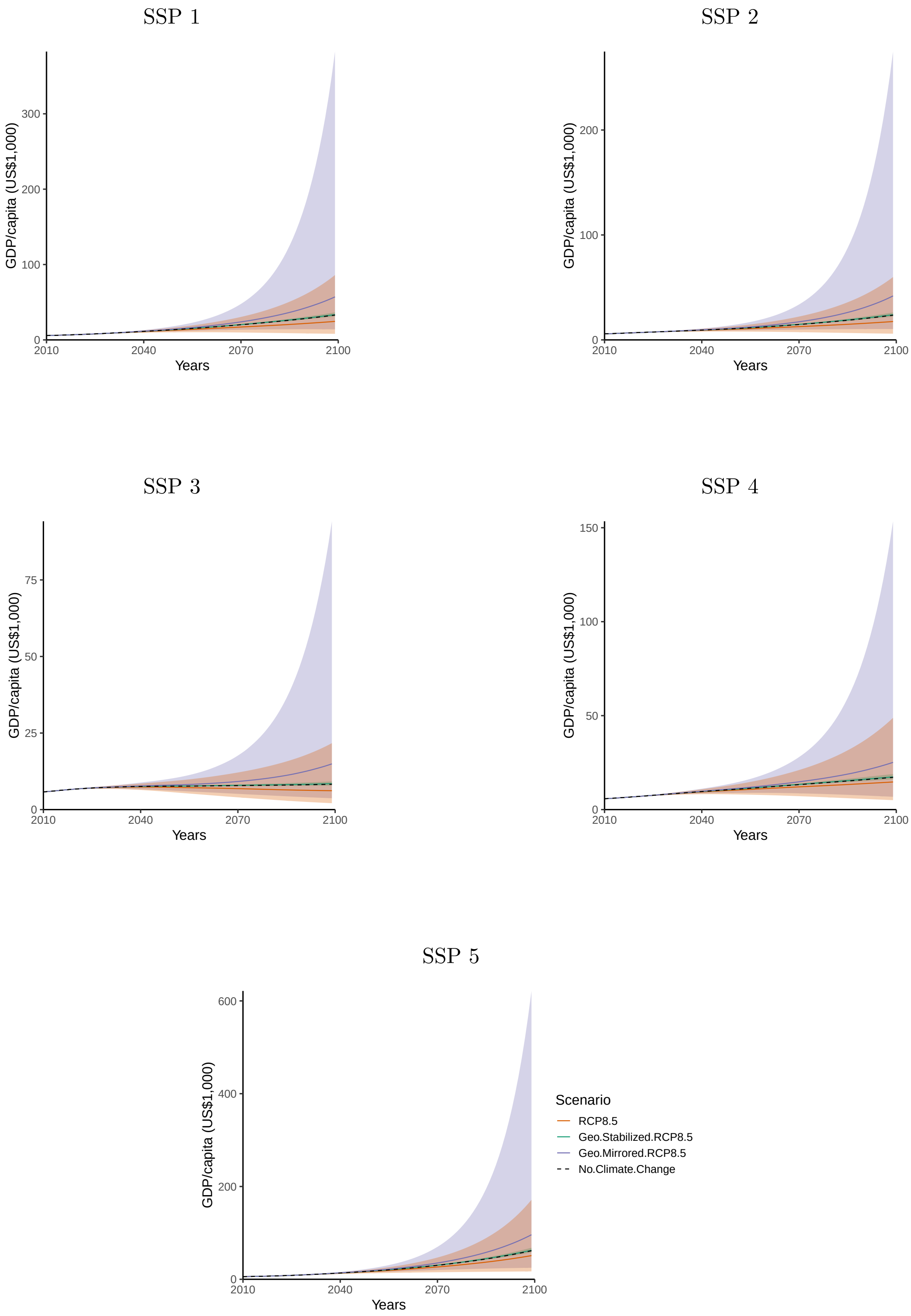
SSP 1
SSP 2
SSP 3
SSP 4
SSP 5
GDP/capita (US$1,000)
Years
2010
2040
2070
2100
0
100
200
300
0
100
200
0
25
50
75
0
50
100
150
0
200
400
600
Scenario
RCP8.5
Geo.Stabilized.RCP8.5
Geo.Mirrored.RCP8.5
No.Climate.Change

c.

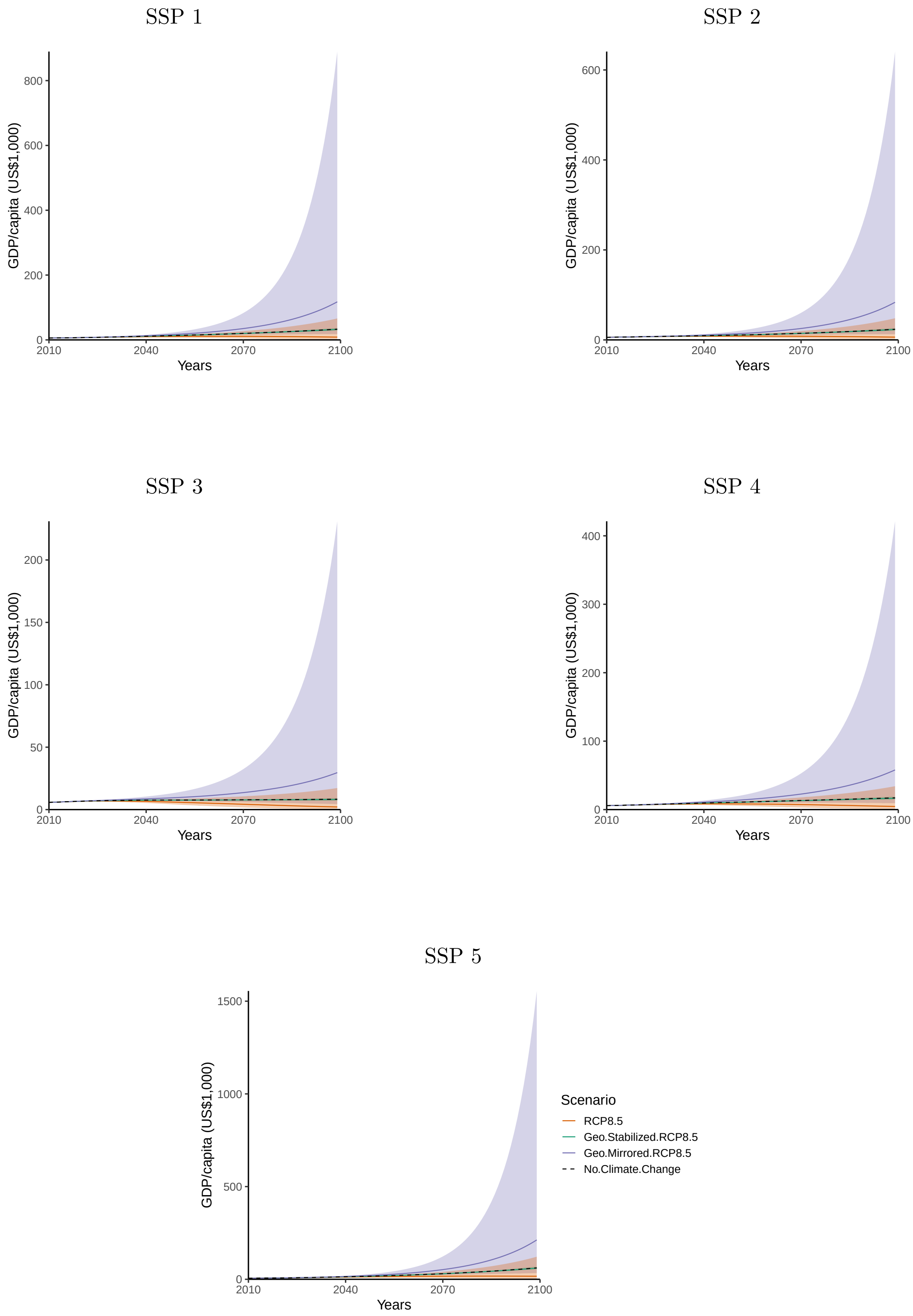
SSP 1
SSP 2
SSP 3
SSP 4
SSP 5
GDP/capita (US$1,000)
Years
2010
2040
2070
2100
Scenario
RCP8.5
Geo.Stabilized.RCP8.5
Geo.Mirrored.RCP8.5
No.Climate.Change

d.

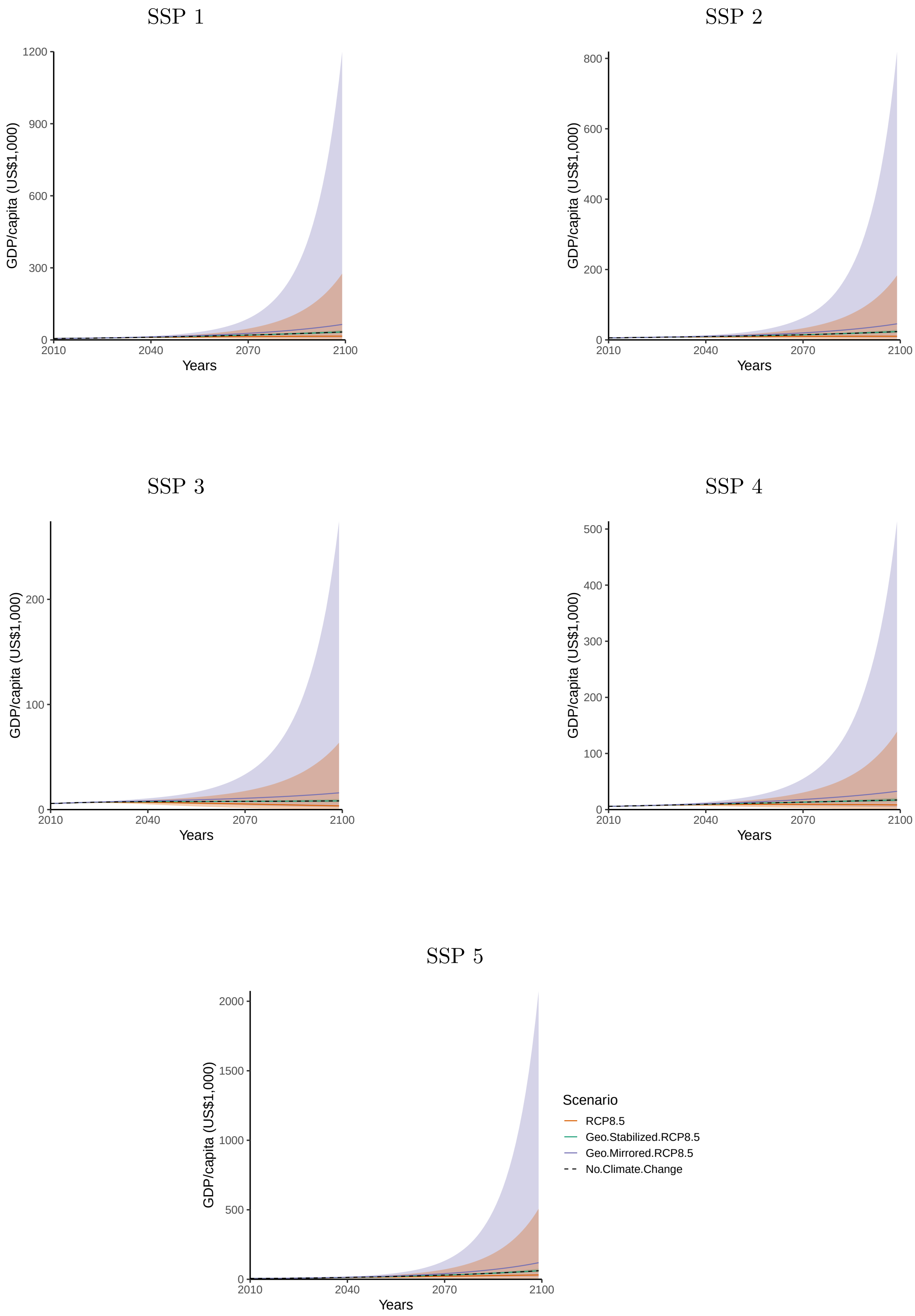
SSP 1
GDP/capita (US$1,000)
0
300
600
900
1200
2010
2040
2070
2100
Years
SSP 2
GDP/capita (US$1,000)
0
200
400
600
800
2010
2040
2070
2100
Years
SSP 3
GDP/capita (US$1,000)
0
100
200
2010
2040
2070
2100
Years
SSP 4
GDP/capita (US$1,000)
0
100
200
300
400
500
2010
2040
2070
2100
Years
SSP 5
GDP/capita (US$1,000)
0
500
1000
1500
2000
2010
2040
2070
2100
Years
Scenario
RCP8.5
Geo.Stabilized.RCP8.5
Geo.Mirrored.RCP8.5
No.Climate.Change

e.

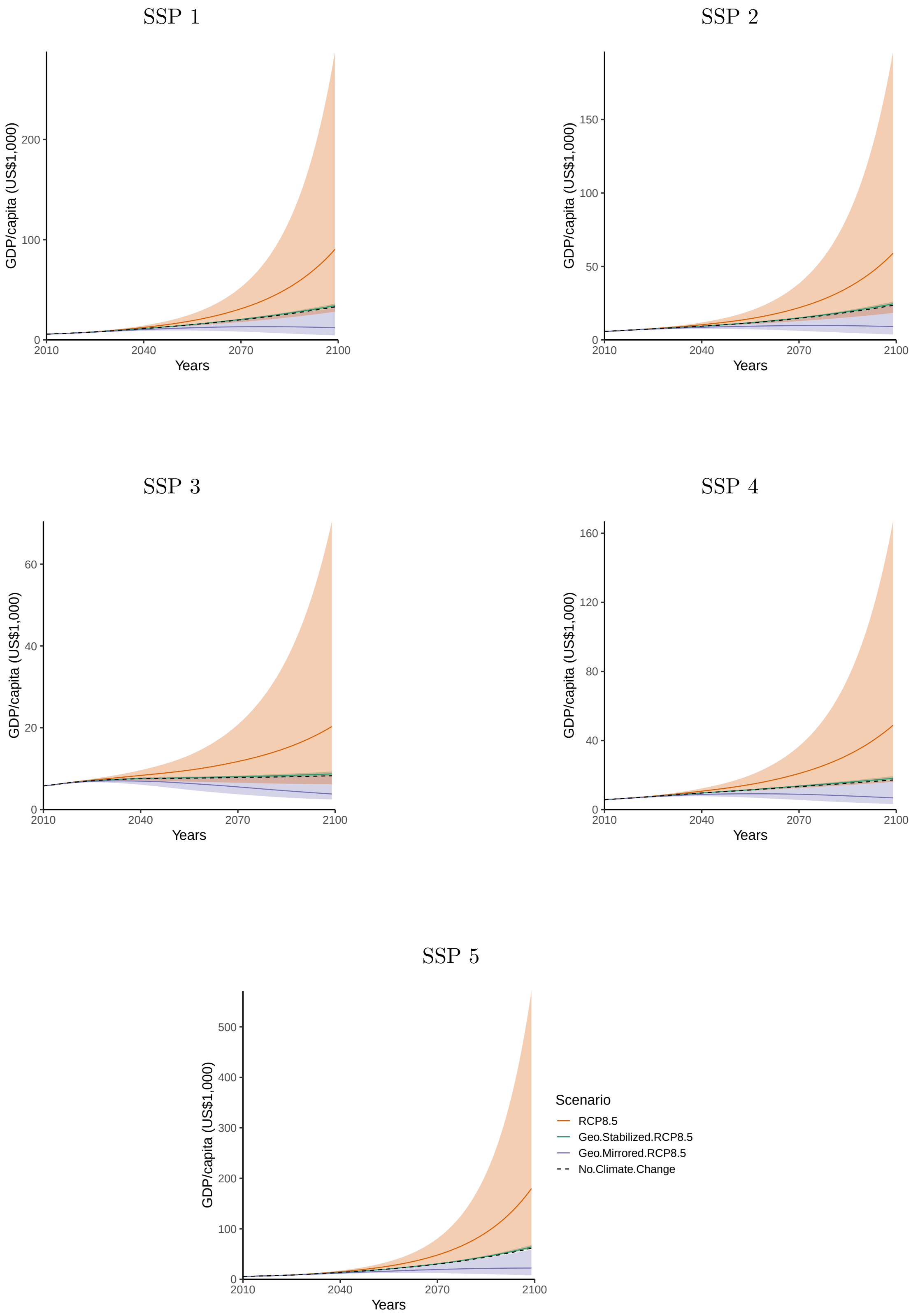

SSP 1
SSP 2
SSP 3
SSP 4
SSP 5
GDP/capita (US$1,000)
Years
2010
2040
2070
2100
Scenario
RCP8.5
Geo.Stabilized.RCP8.5
Geo.Mirrored.RCP8.5
No.Climate.Change

f.

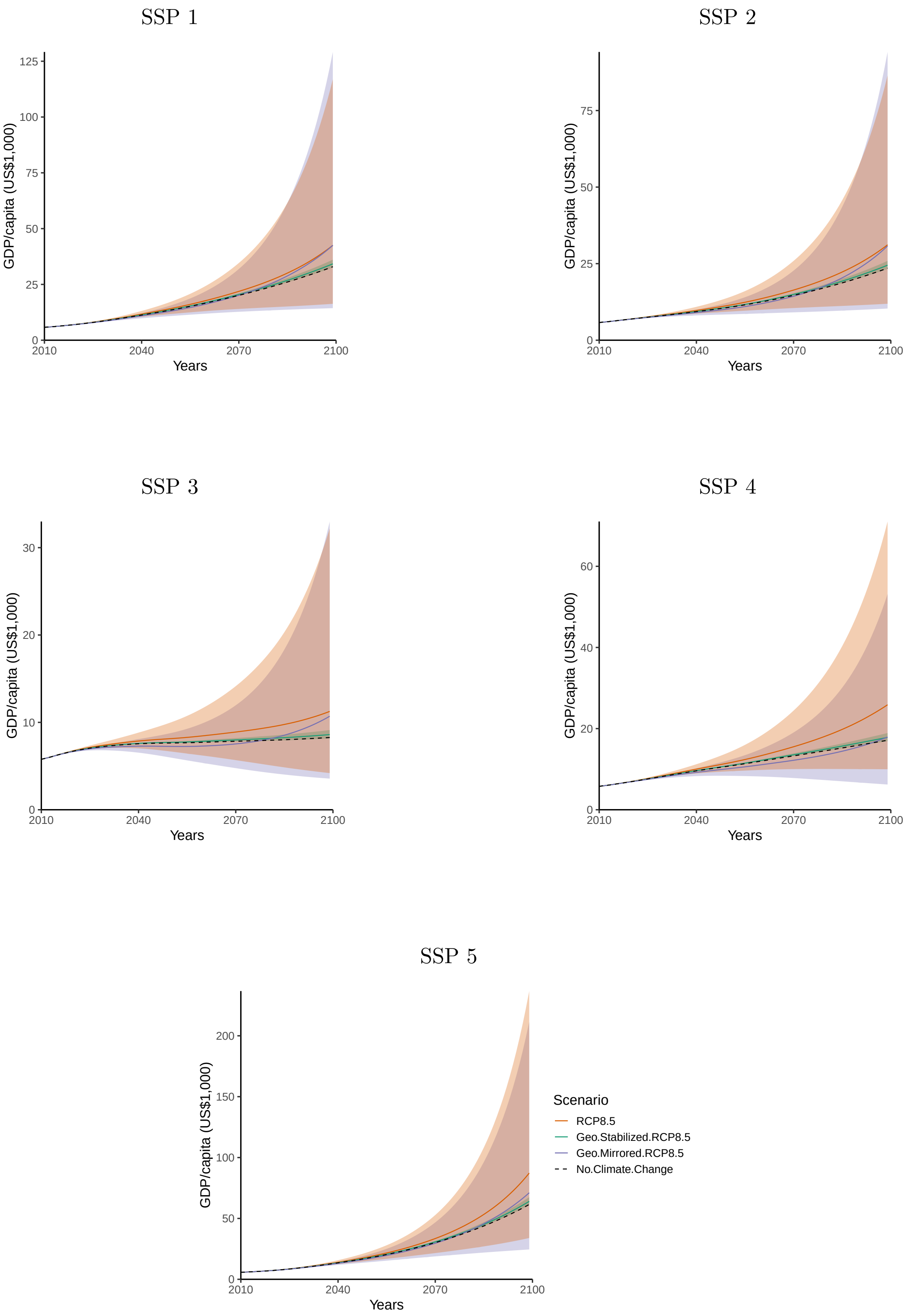
SSP 1
SSP 2
SSP 3
SSP 4
SSP 5
GDP/capita (US$1,000)
Years
2010
2040
2070
2100
Scenario
RCP8.5
Geo.Stabilized.RCP8.5
Geo.Mirrored.RCP8.5
No.Climate.Change

g.

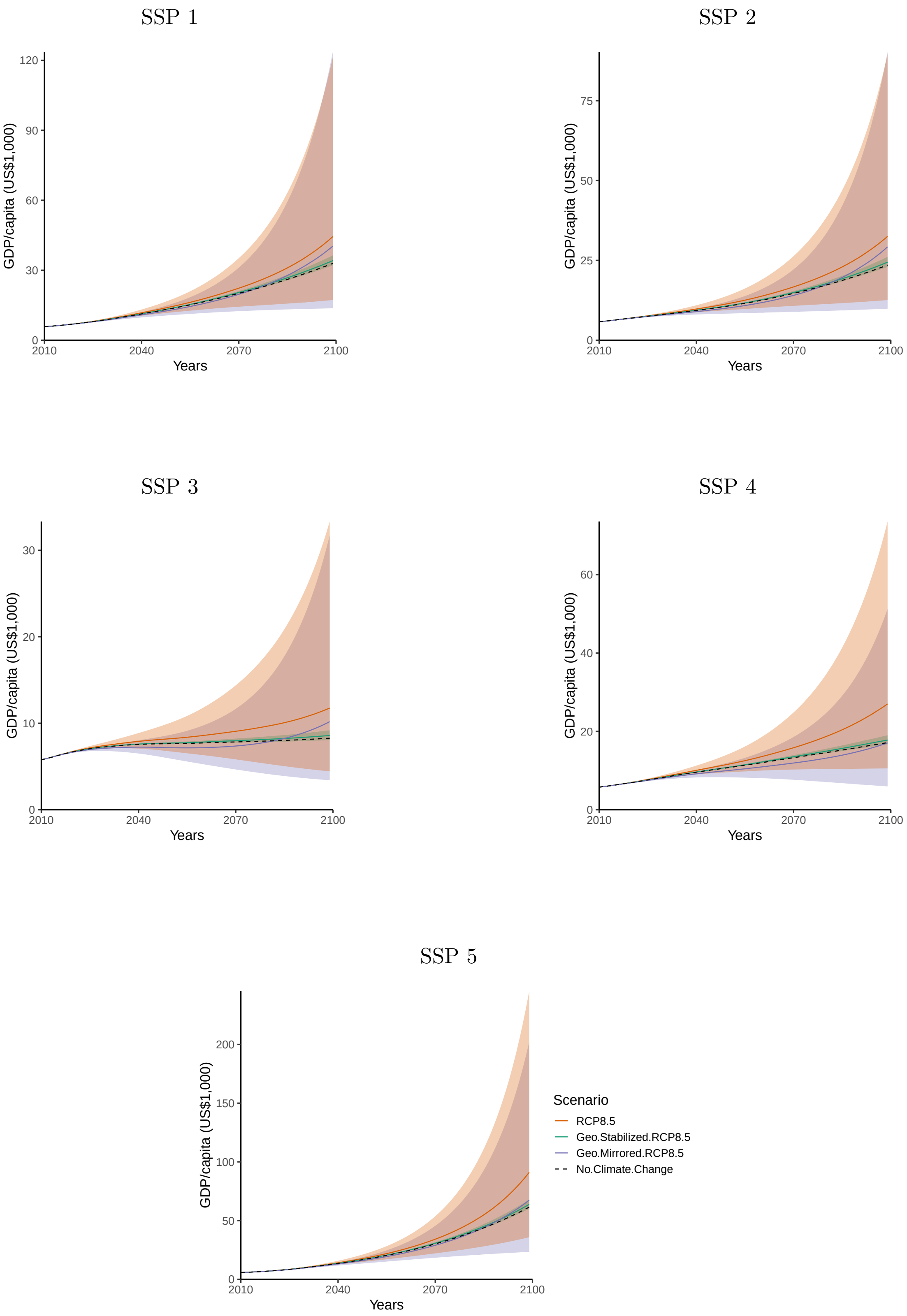
SSP 1
SSP 2
SSP 3
SSP 4
SSP 5
GDP/capita (US$1,000)
Years
2010
2040
2070
2100
0
30
60
90
120
25
50
75
10
20
40
150
100
200
Scenario
RCP8.5
Geo.Stabilized.RCP8.5
Geo.Mirrored.RCP8.5
No.Climate.Change

h.

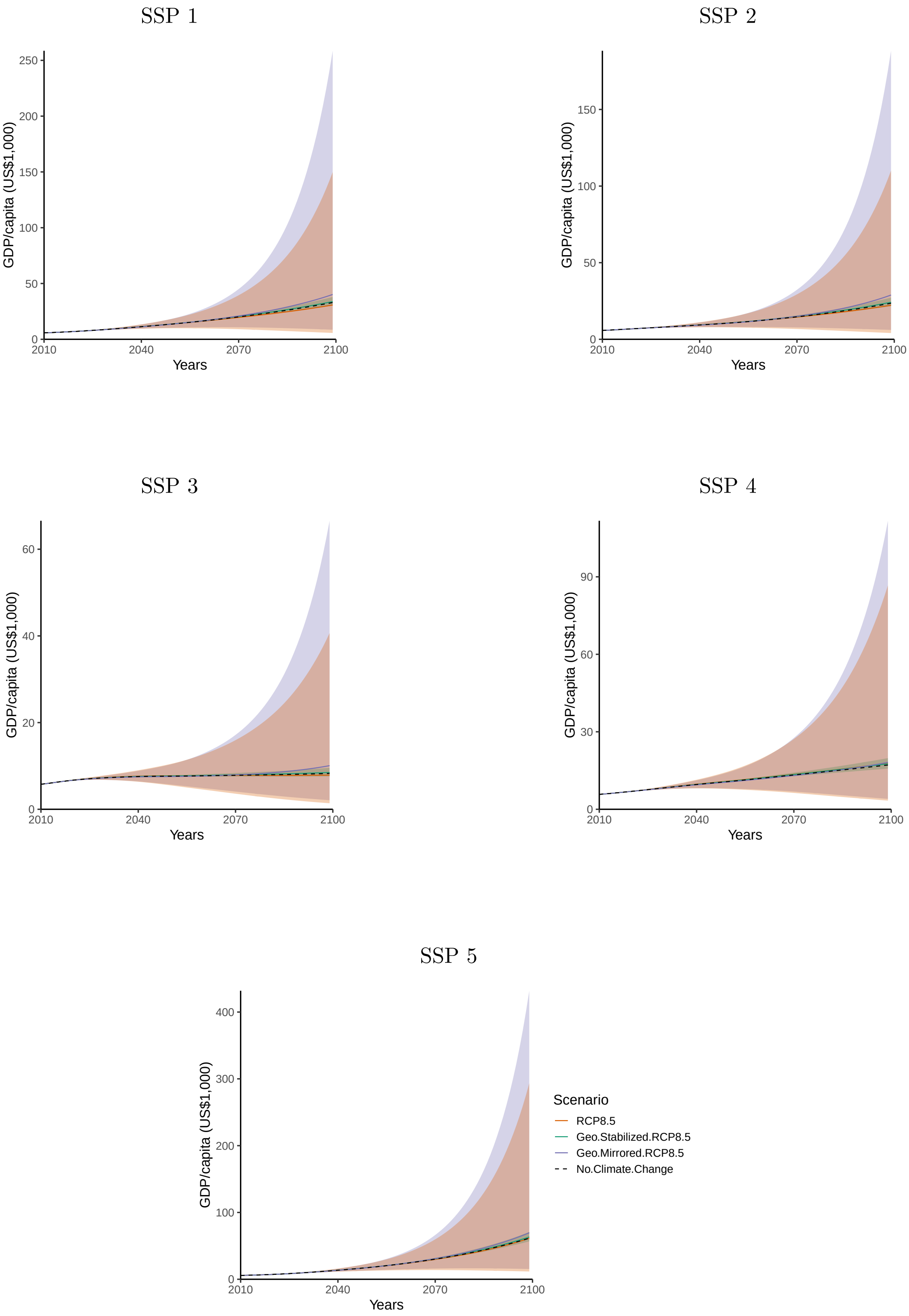
SSP 1
SSP 2
SSP 3
SSP 4
SSP 5
GDP/capita (US$1,000)
Years
0
50
100
150
200
250
300
400
20
40
60
30
90
2010
2040
2070
2100
Scenario
RCP8.5
Geo.Stabilized.RCP8.5
Geo.Mirrored.RCP8.5
No.Climate.Change

i.

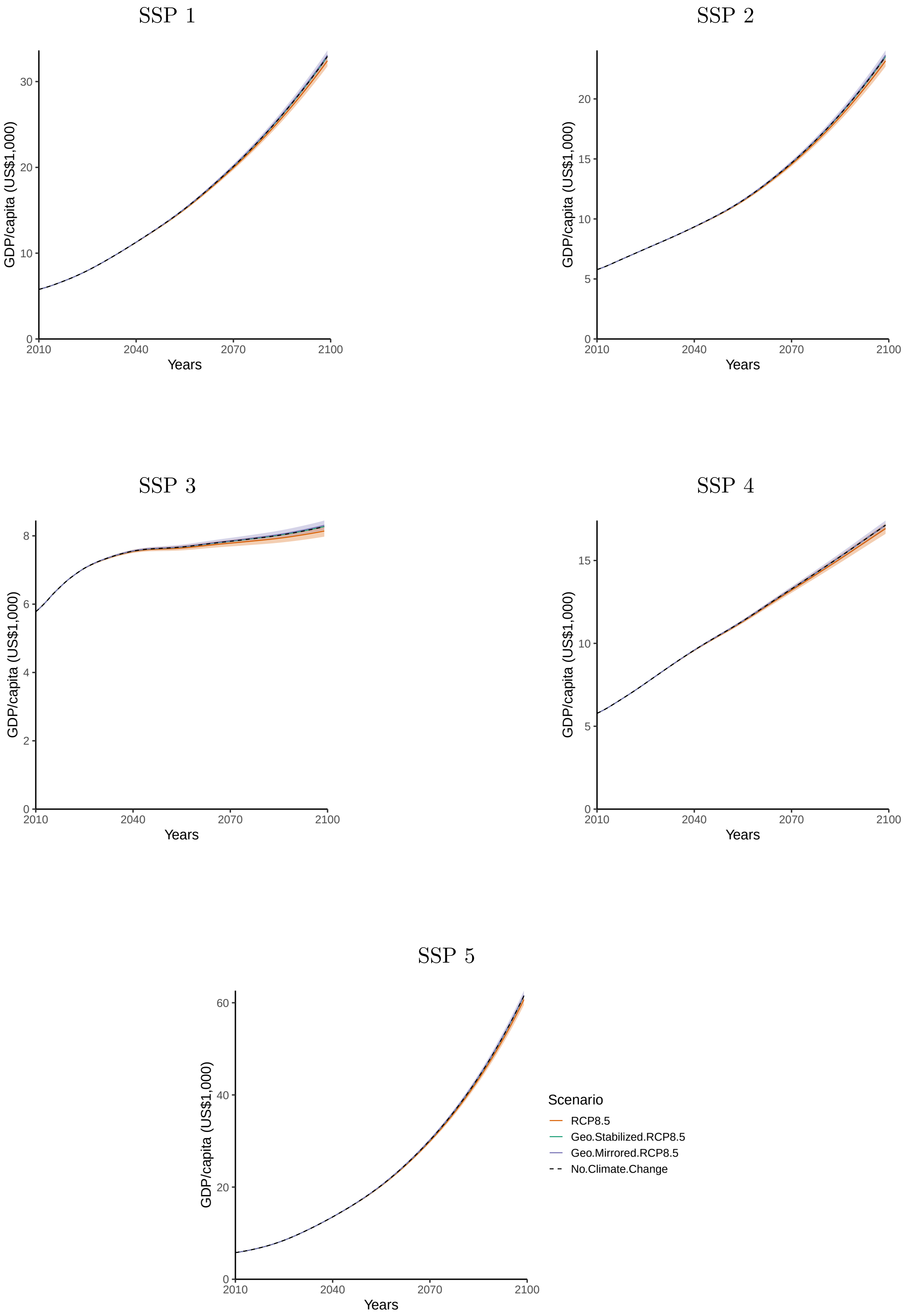
SSP 1
SSP 2
SSP 3
SSP 4
SSP 5
GDP/capita (US$1,000)
Years
2010
2040
2070
2100
Scenario
RCP8.5
Geo.Stabilized.RCP8.5
Geo.Mirrored.RCP8.5
No.Climate.Change

j.

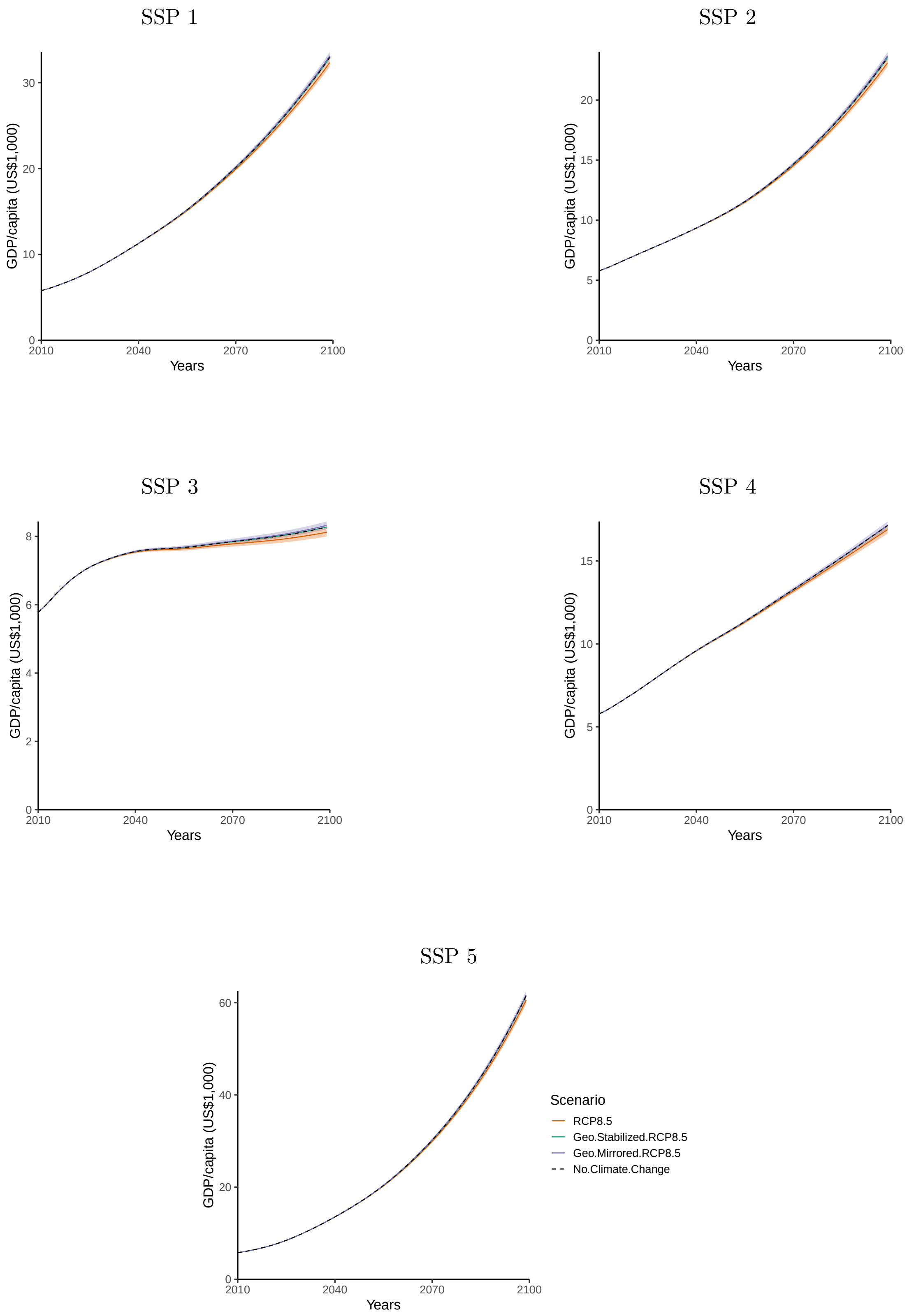
SSP 1
SSP 2
SSP 3
SSP 4
SSP 5
GDP/capita (US$1,000)
Years
2010
2040
2070
2100
Scenario
RCP8.5
Geo.Stabilized.RCP8.5
Geo.Mirrored.RCP8.5
No.Climate.Change

k.

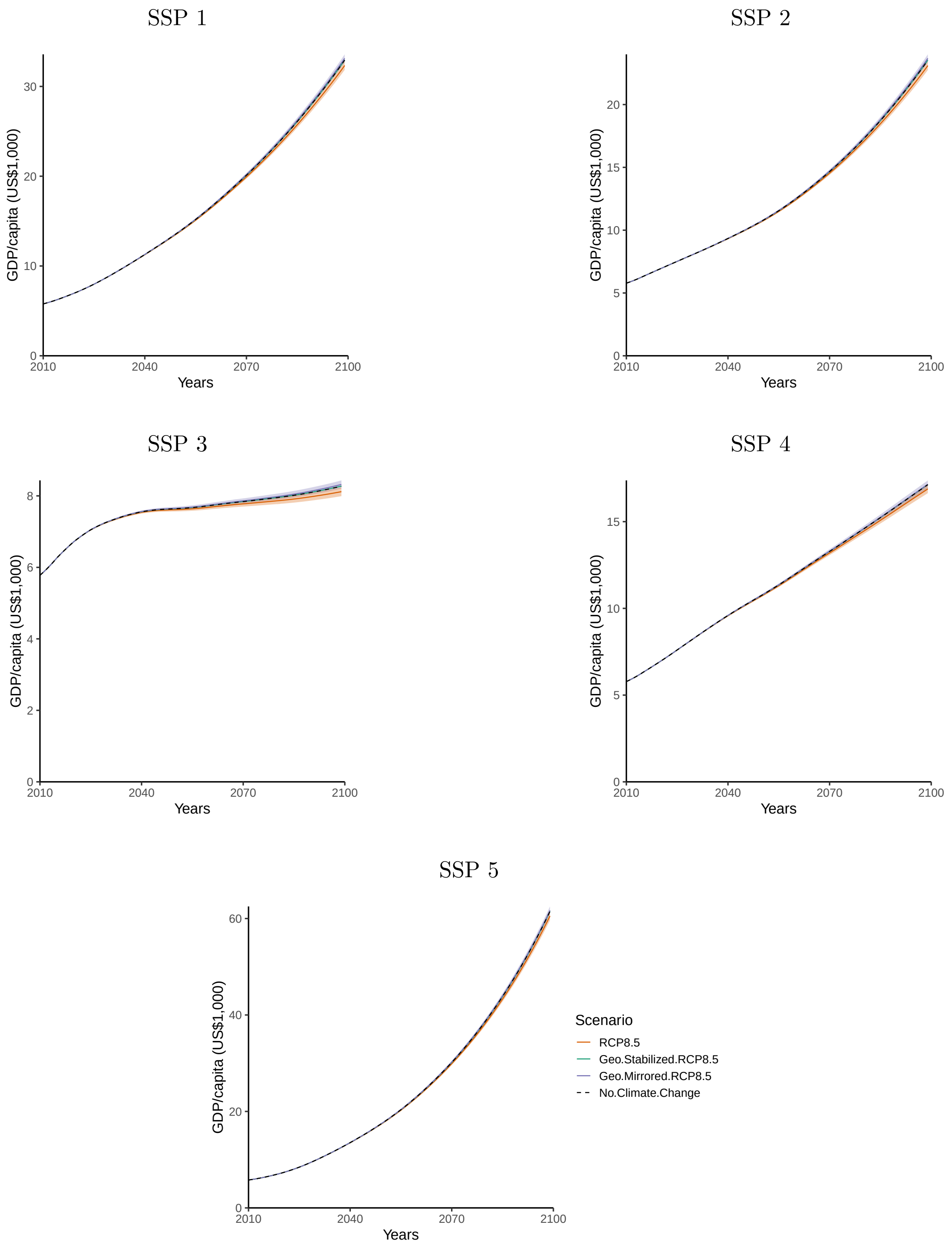

Supplementary Figure 2 | Projected global GDP per capita over the 21st Century. Each line represents the median projection different climate scenario. The filled area represents the 95% confidence interval for climate impacts. Each panel represents the projections from a different Shared Socio-economic Pathway (SSP). **a** uses the model from column (1) in Table S1; **b** uses column (2); **c** uses column (3); **d** uses column (4); **e** uses column (5); **f** uses column (6); **g** uses column (7); **h** uses column (8); **i** uses column (9); **j** uses column (10); **k** uses column (11).

a.

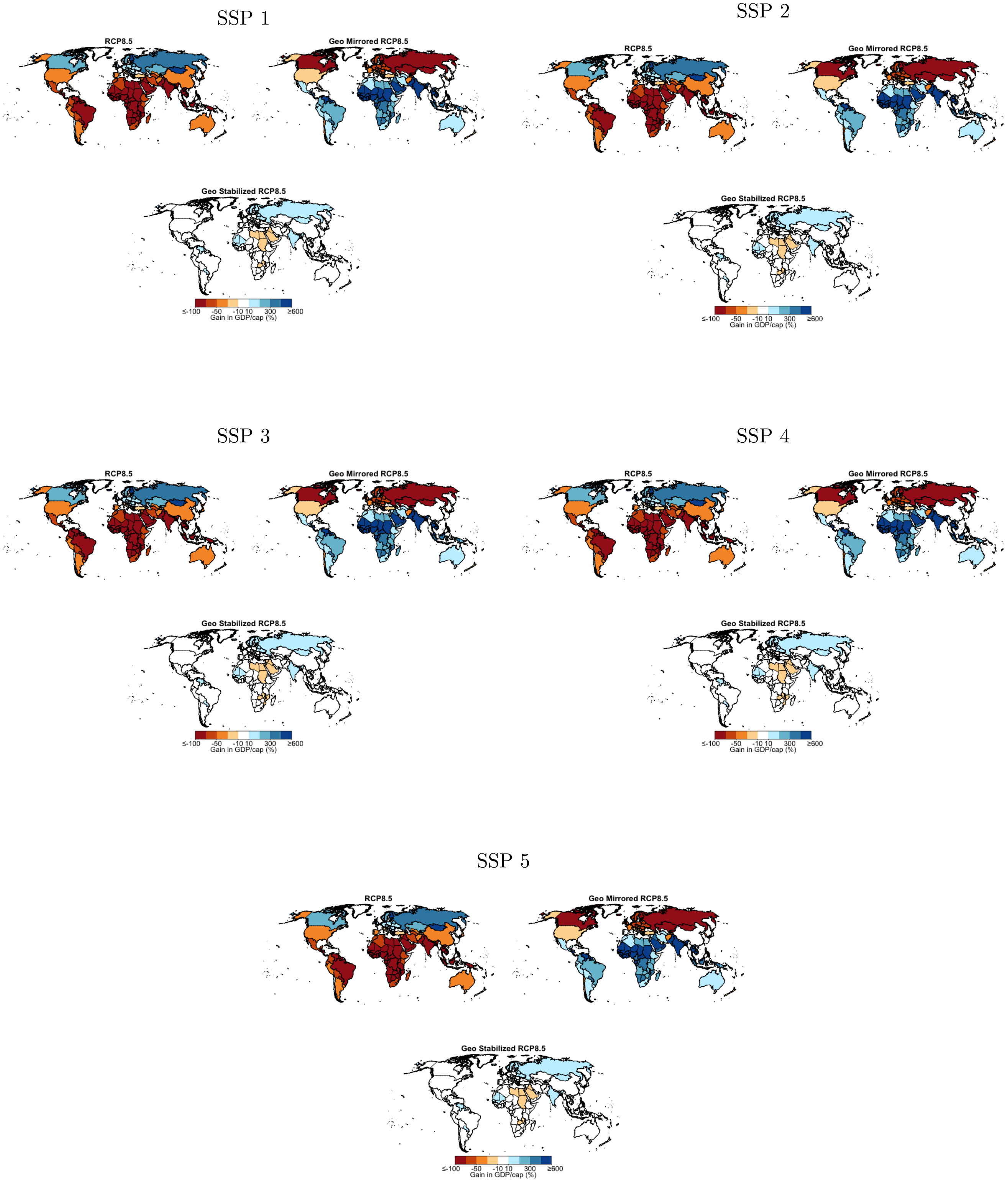
SSP 1
RCP8.5
Geo Mirrored RCP8.5
Geo Stabilized RCP8.5
≤-100 -50 -10 10 300 ≥600
Gain in GDP/cap (%)
SSP 2
RCP8.5
Geo Mirrored RCP8.5
Geo Stabilized RCP8.5
≤-100 -50 -10 10 300 ≥600
Gain in GDP/cap (%)
SSP 3
RCP8.5
Geo Mirrored RCP8.5
Geo Stabilized RCP8.5
≤-100 -50 -10 10 300 ≥600
Gain in GDP/cap (%)
SSP 4
RCP8.5
Geo Mirrored RCP8.5
Geo Stabilized RCP8.5
≤-100 -50 -10 10 300 ≥600
Gain in GDP/cap (%)
SSP 5
RCP8.5
Geo Mirrored RCP8.5
Geo Stabilized RCP8.5
≤-100 -50 -10 10 300 ≥600
Gain in GDP/cap (%)

b.

SSP 1

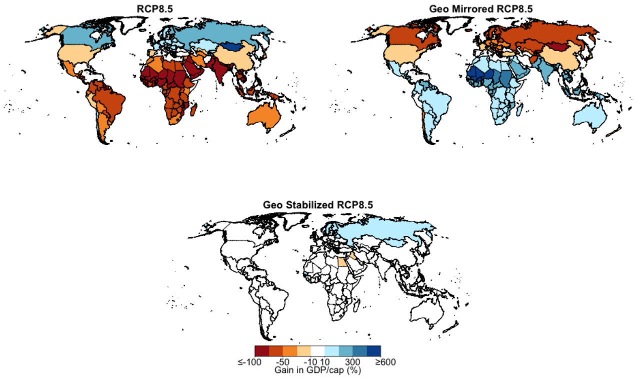

SSP 2

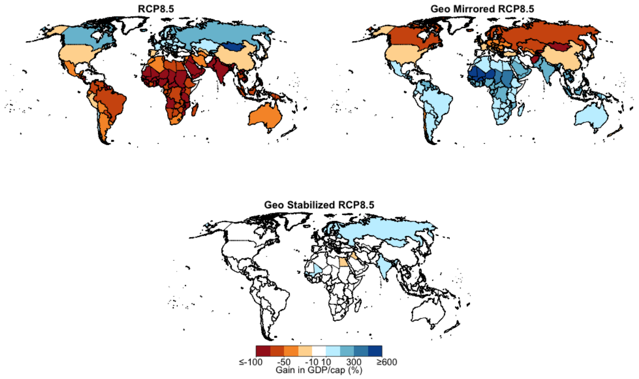

SSP 3

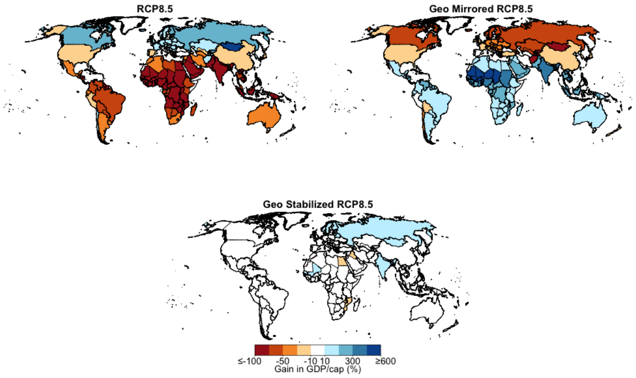

SSP 4

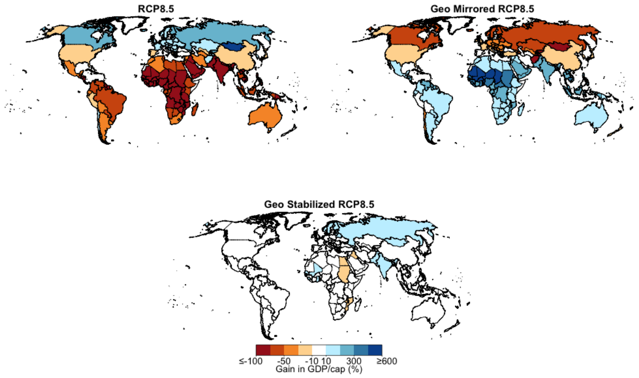

SSP 5

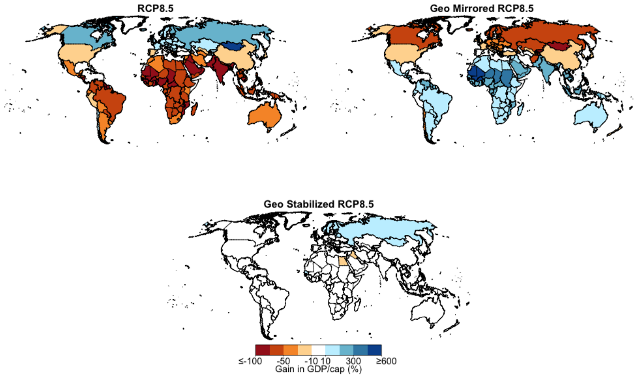

c.

SSP 1

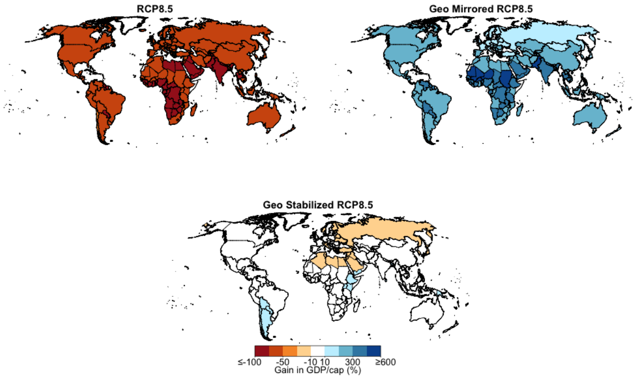

SSP 2

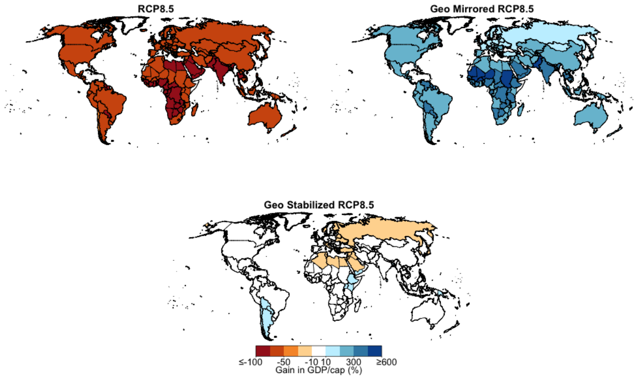

SSP 3

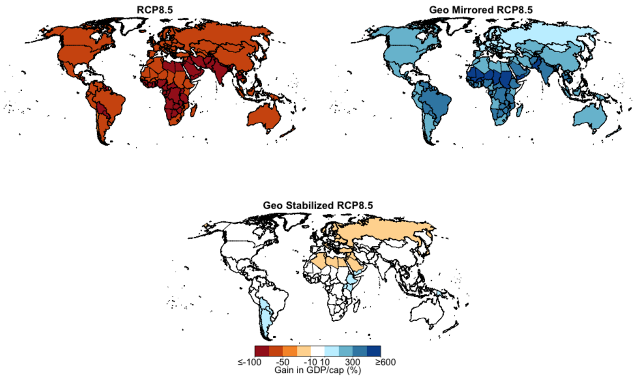

SSP 4

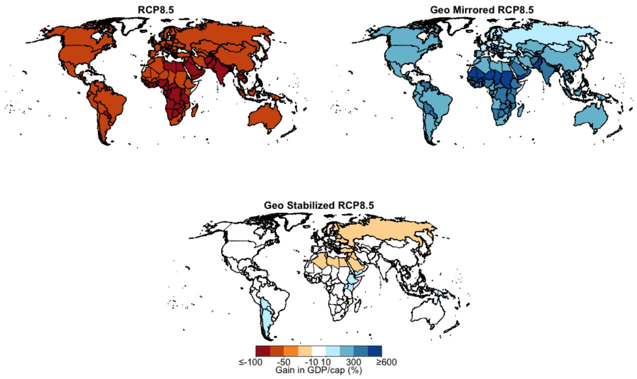

SSP 5

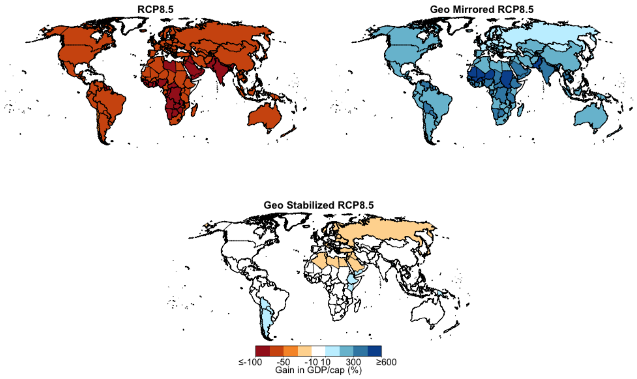

d.

SSP 1

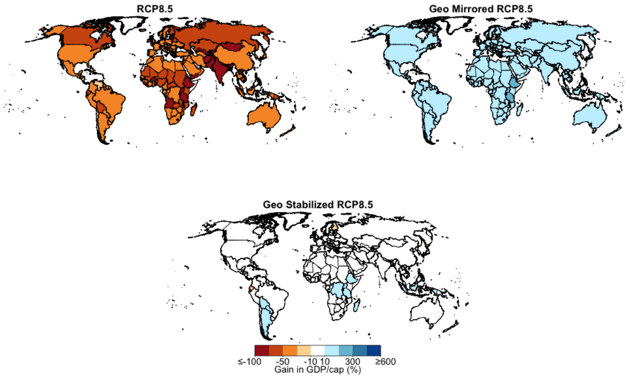

SSP 2

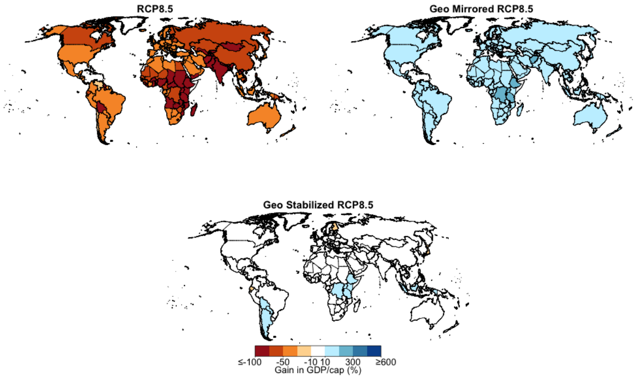

SSP 3

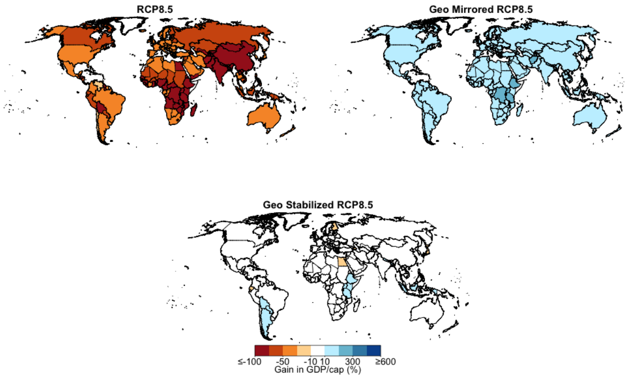

SSP 4

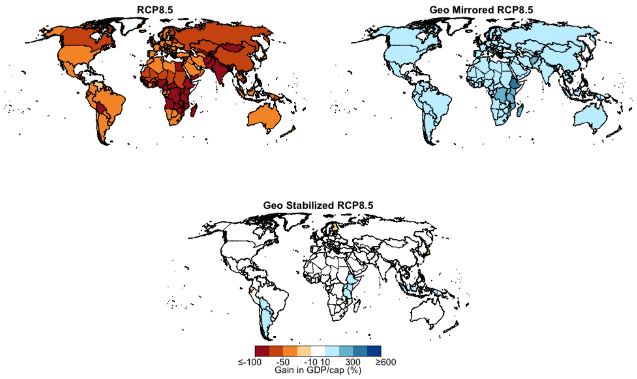

SSP 5

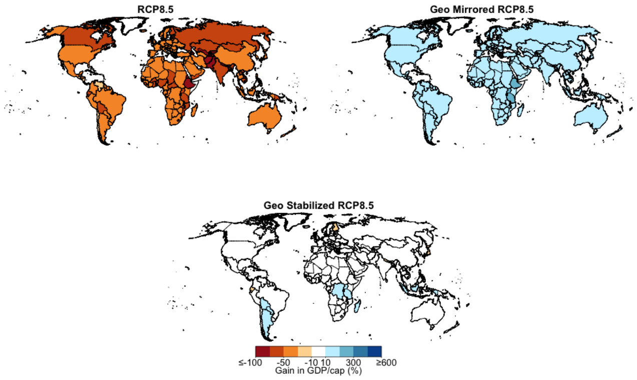

e.

SSP 1

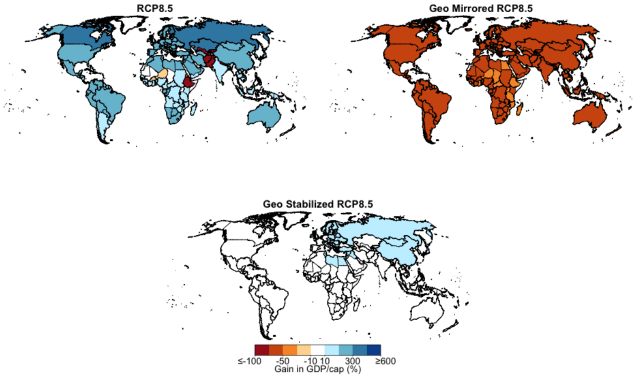

SSP 2

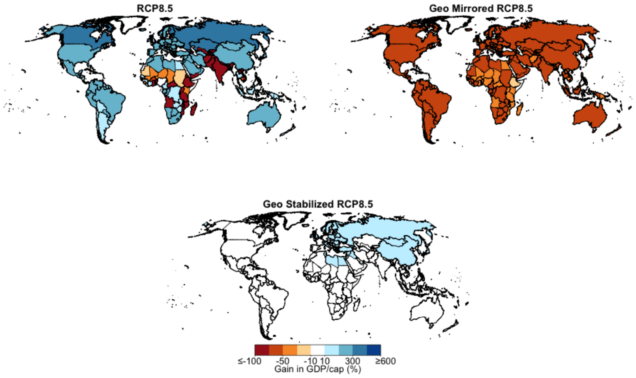

SSP 3

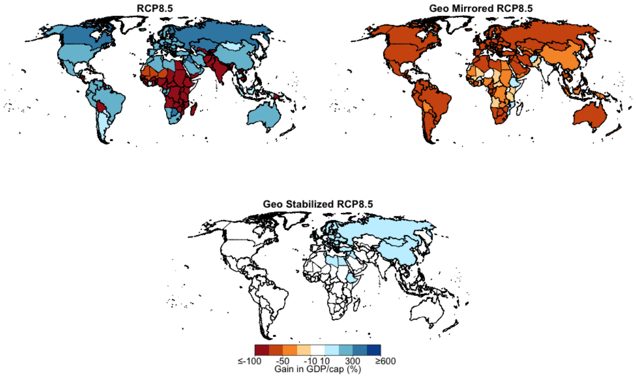

SSP 4

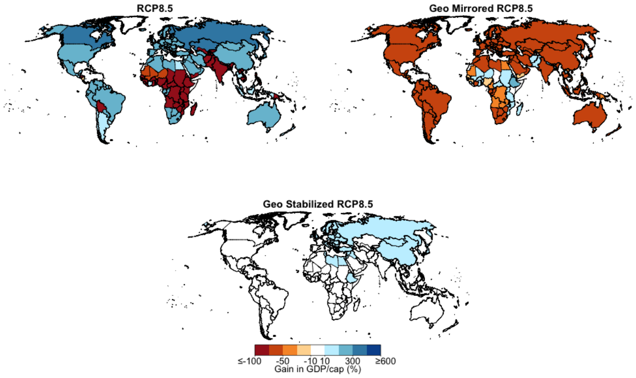

SSP 5

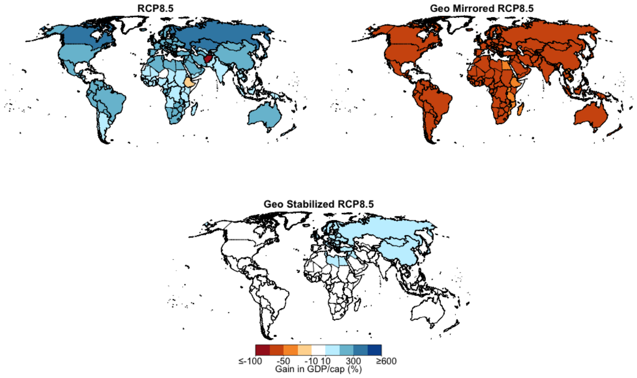

f.

SSP 1

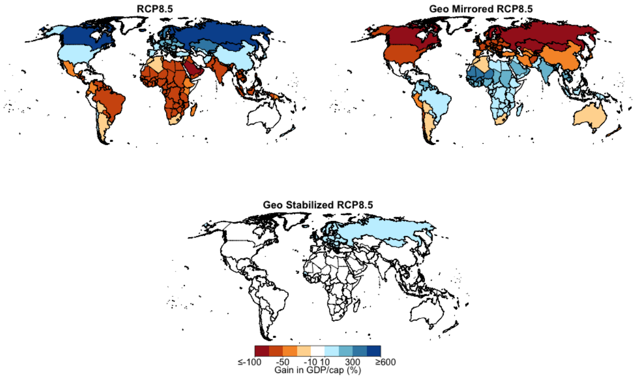

SSP 2

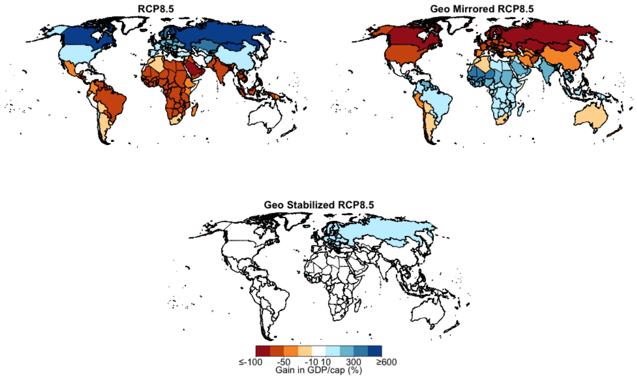

SSP 3

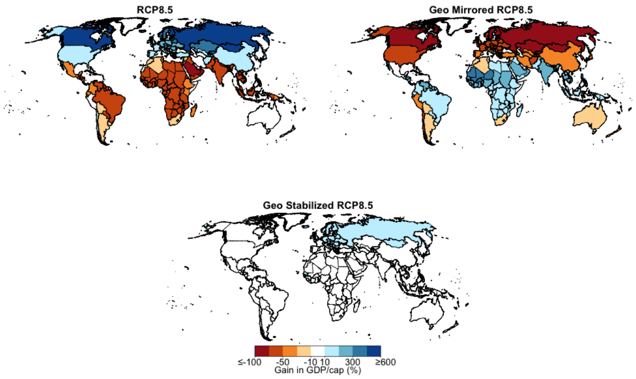

SSP 4

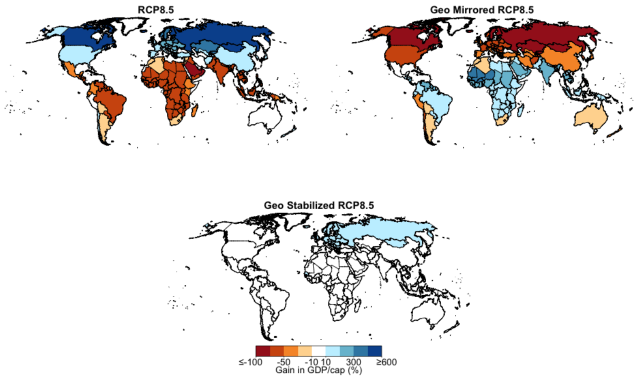

SSP 5

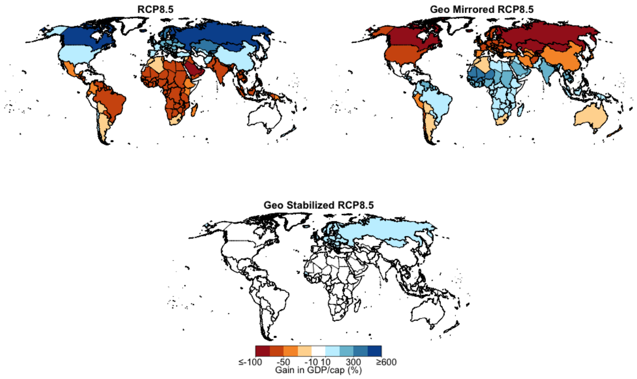

g.

SSP 1

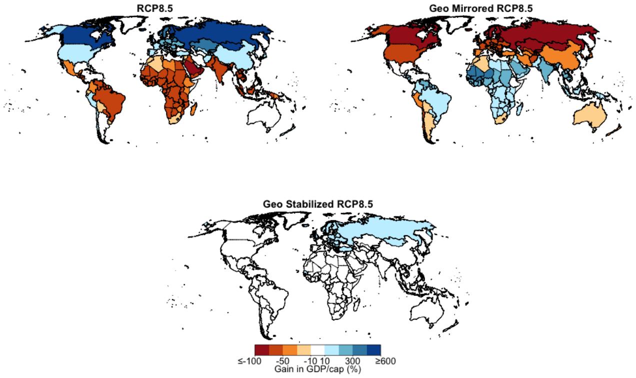

SSP 2

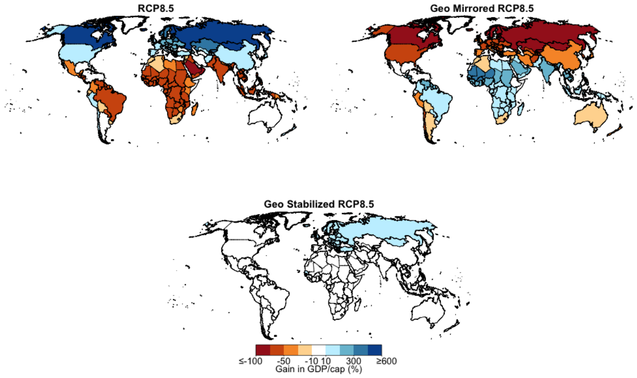

SSP 3

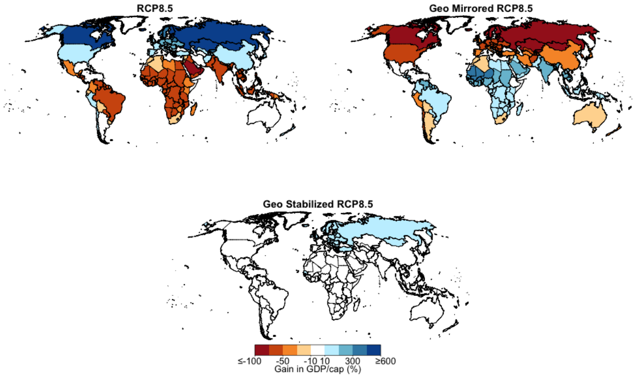

SSP 4

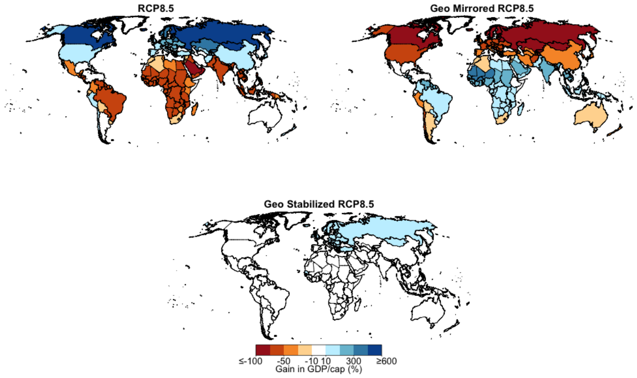

SSP 5

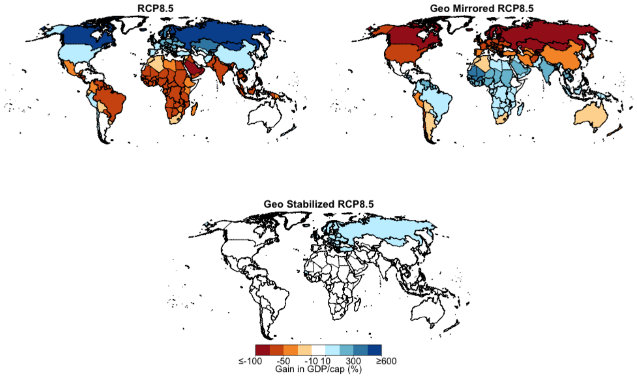

h.

SSP 1

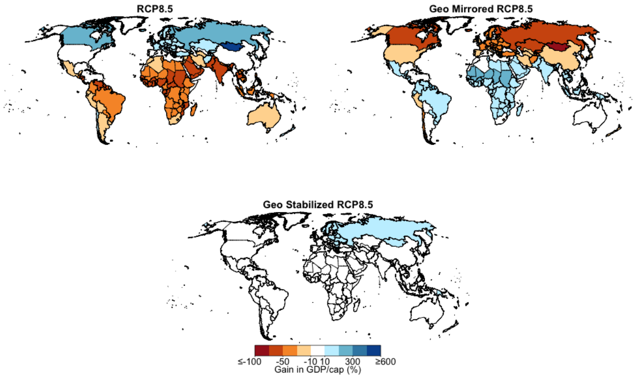

SSP 2

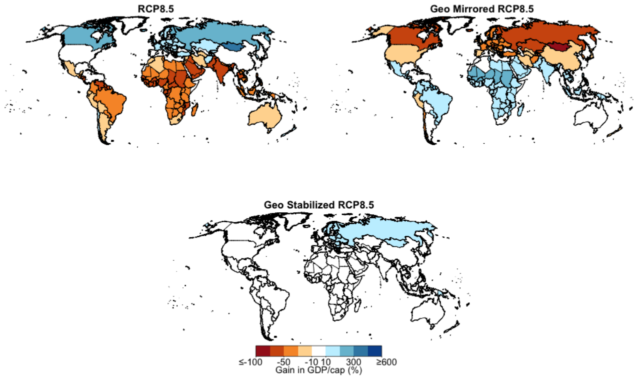

SSP 3

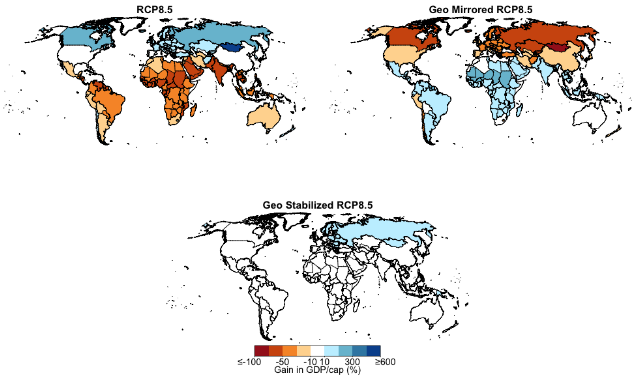

SSP 4

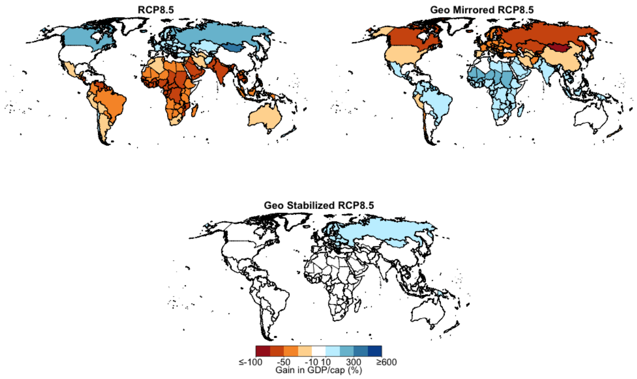

SSP 5

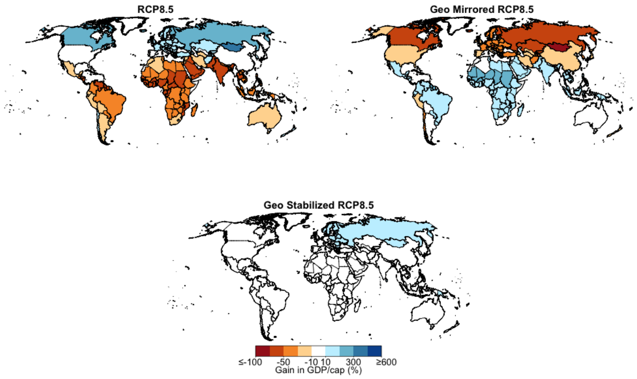

i.

SSP 1

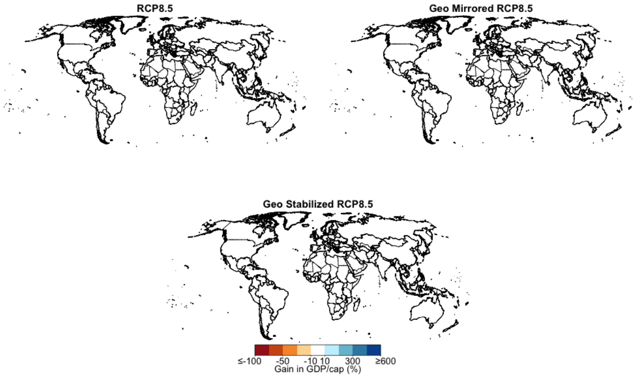

SSP 2

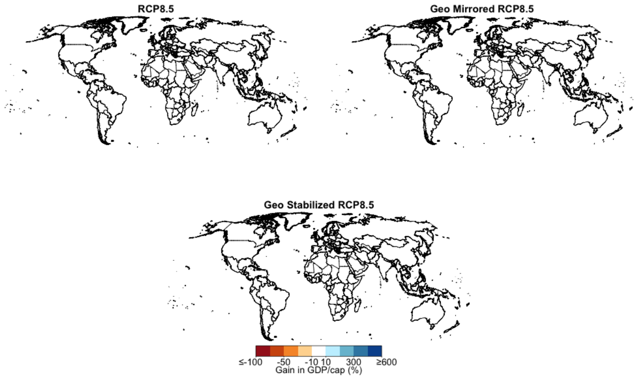

SSP 3

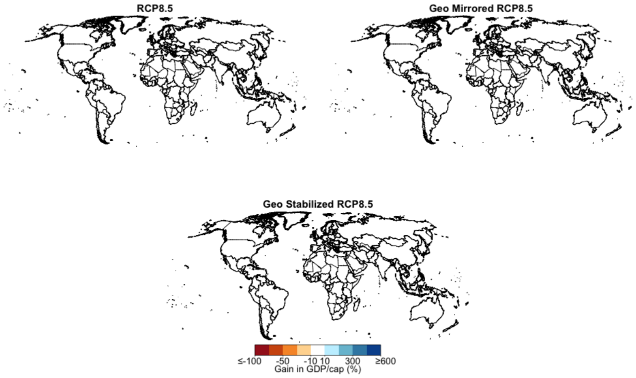

SSP 4

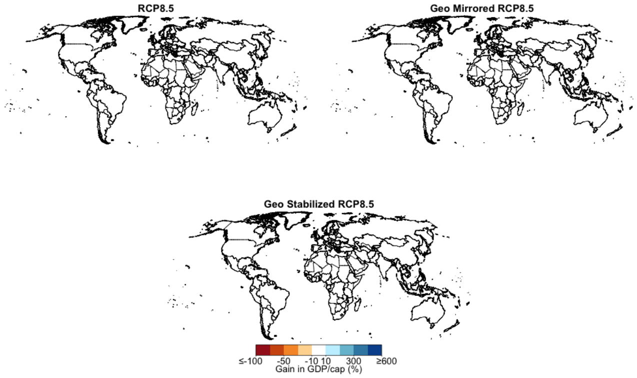

SSP 5

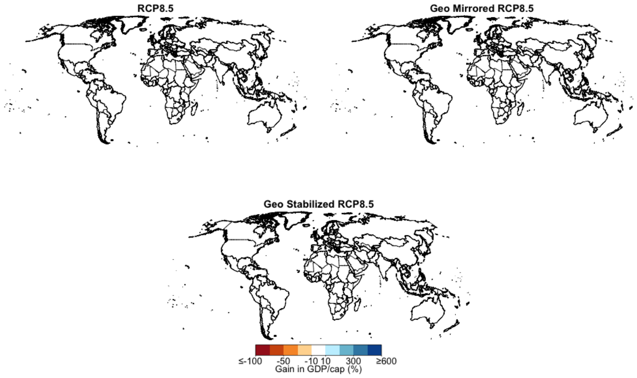

j.

SSP 1

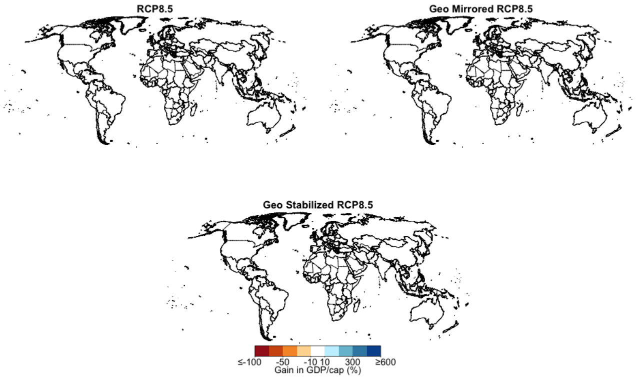

SSP 2

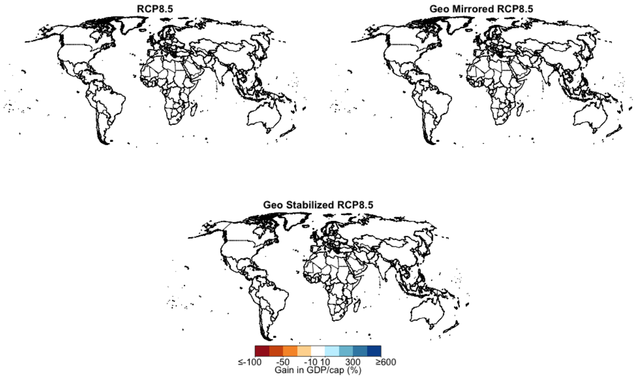

SSP 3

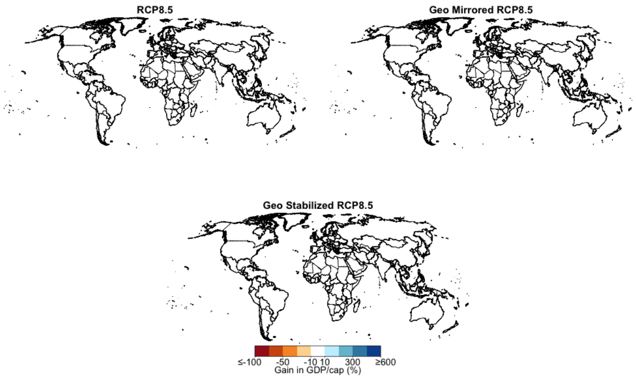

SSP 4

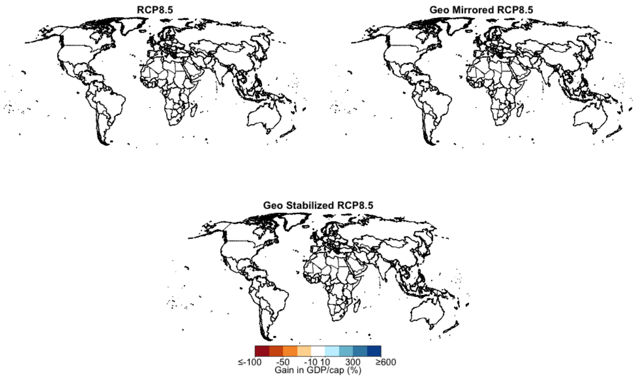

SSP 5

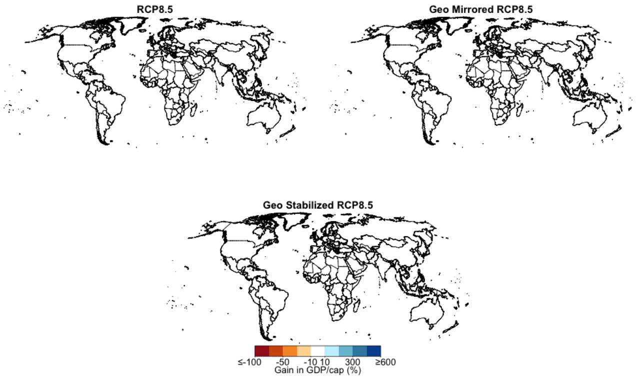

k.

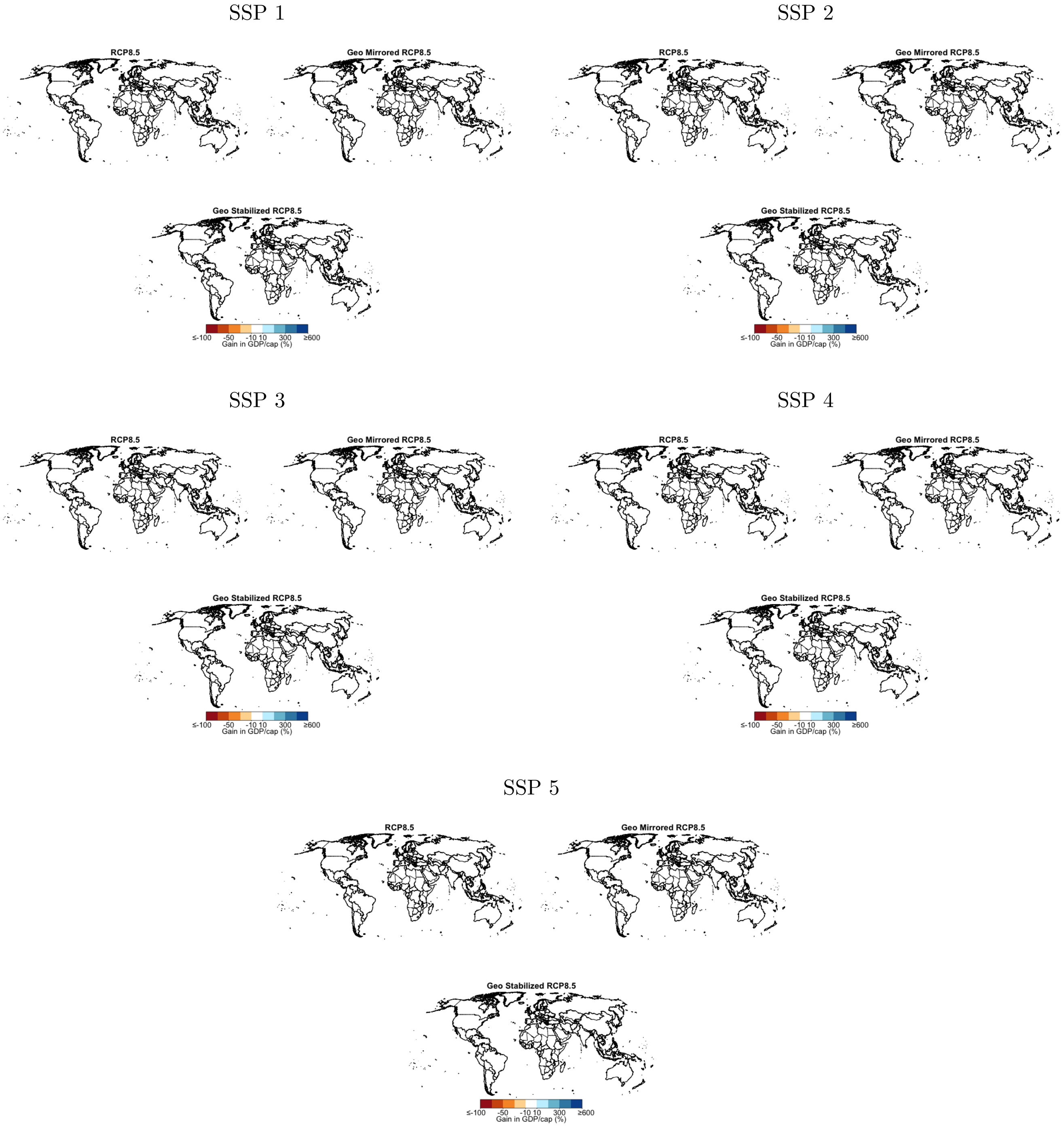

Supplementary Figure 3 | Percentage Gain in GDP per capita in 2099 relative to the scenario with no changes. Each panel displays the country-level percentage gain in GDP per capita for the RCP 8.5, geoengineering mirrored RCP 8.5, and geoengineering stabilized RCP 8.5 scenarios relative no changes scenario in 2099. **a** uses the model from column (1) in Table S1; **b** uses column (2); **c** uses column (3); **d** uses column (4); **e** uses column (5); **f** uses column (6); **g** uses column (7); **h** uses column (8); **i** uses column (9); **j** uses column (10); **k** uses column (11).

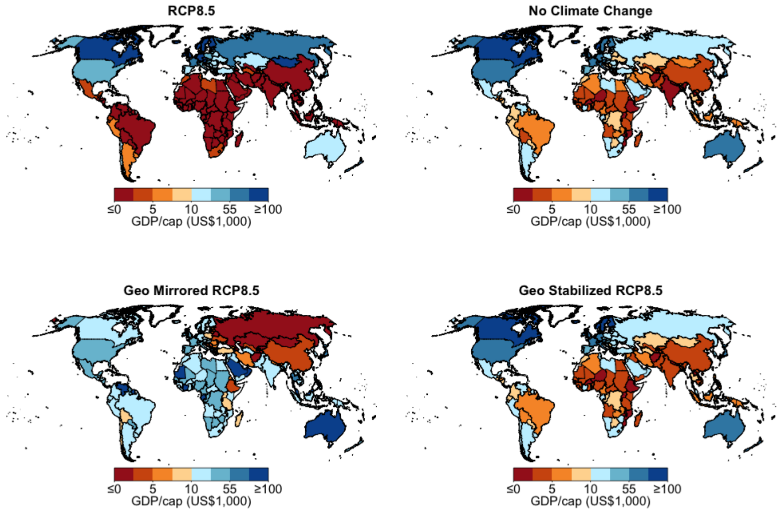

Supplementary Figure 4 | GDP per capita in 2099. Projected country-level GDP per capita in 2099. Results are for the model in column (1) of Table S1 for SSP3.

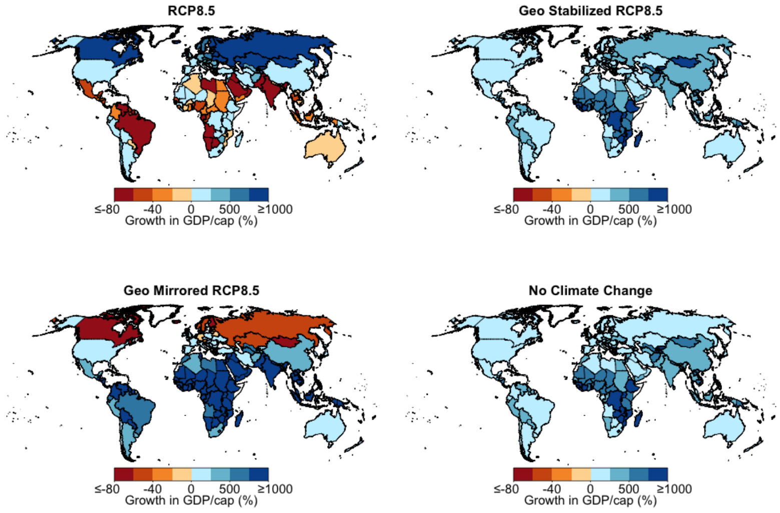

Supplementary Figure 5 | Percentage Gain in GDP per capita from 2010 to 2099. Results are for the model in column (1) of Table S1 for SSP3.

a.

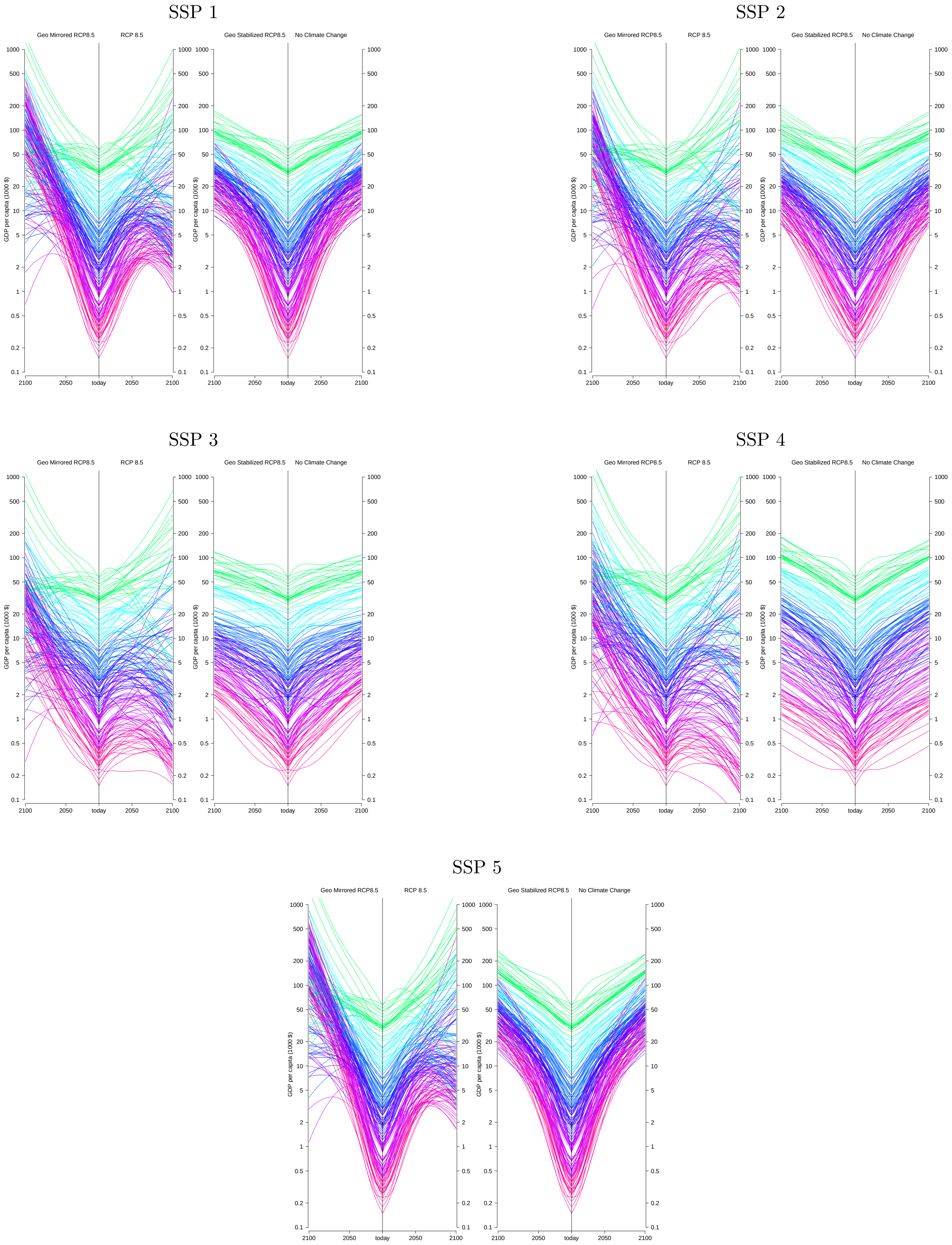

SSP 1
SSP 2
SSP 3
SSP 4
SSP 5
Geo Mirrored RCP8.5
RCP 8.5
Geo Stabilized RCP8.5
No Climate Change
GDP per capita (1000 $)
1000
500
200
100
50
20
10
5
2
1
0.5
0.2
0.1
2100
2050
today
2050
2100

b.

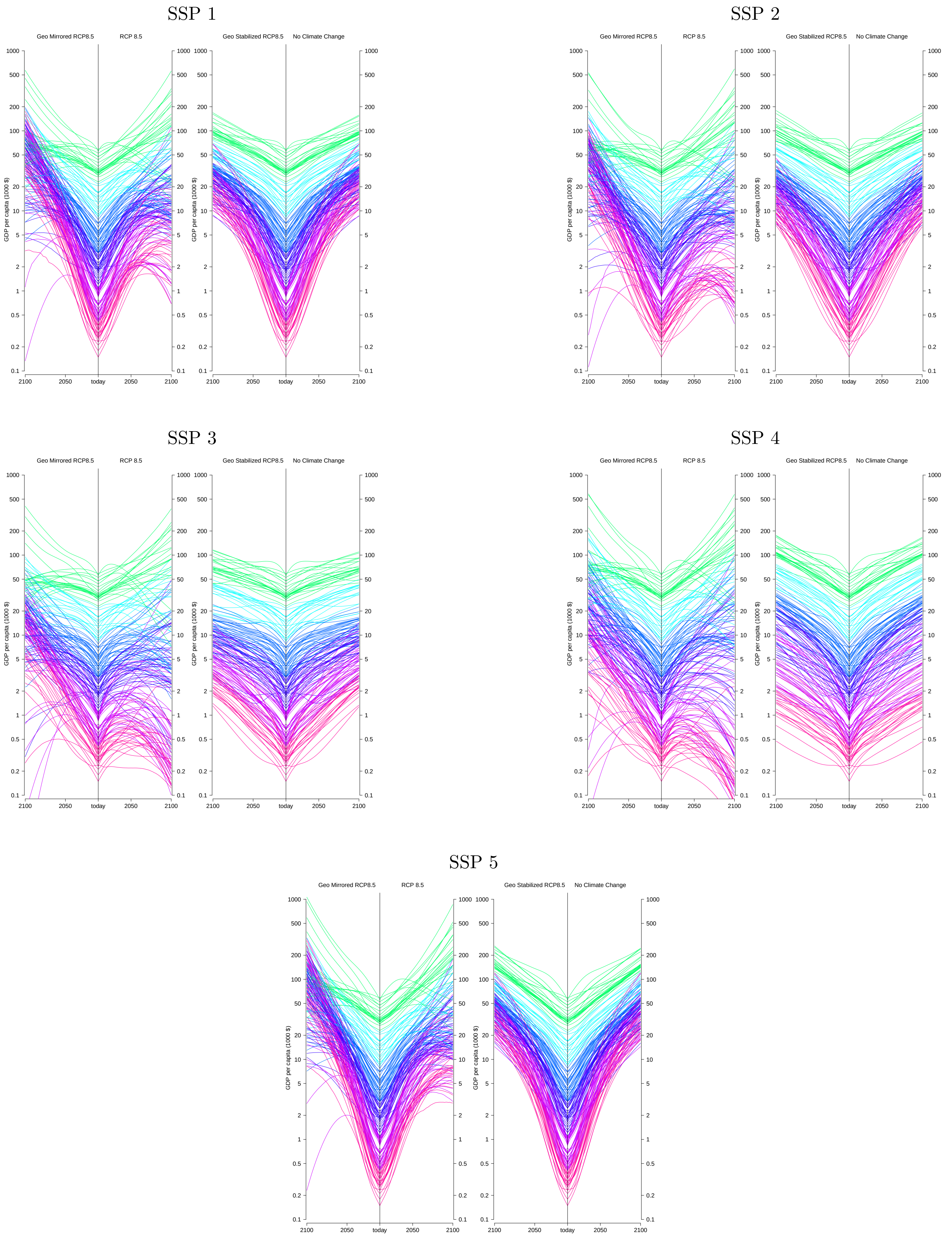
SSP 1
SSP 2
SSP 3
SSP 4
SSP 5
Geo Mirrored RCP8.5
RCP 8.5
Geo Stabilized RCP8.5
No Climate Change
GDP per capita (1000 $)
1000
500
200
100
50
20
10
5
2
1
0.5
0.2
0.1
2100
2050
today
2050
2100

c.

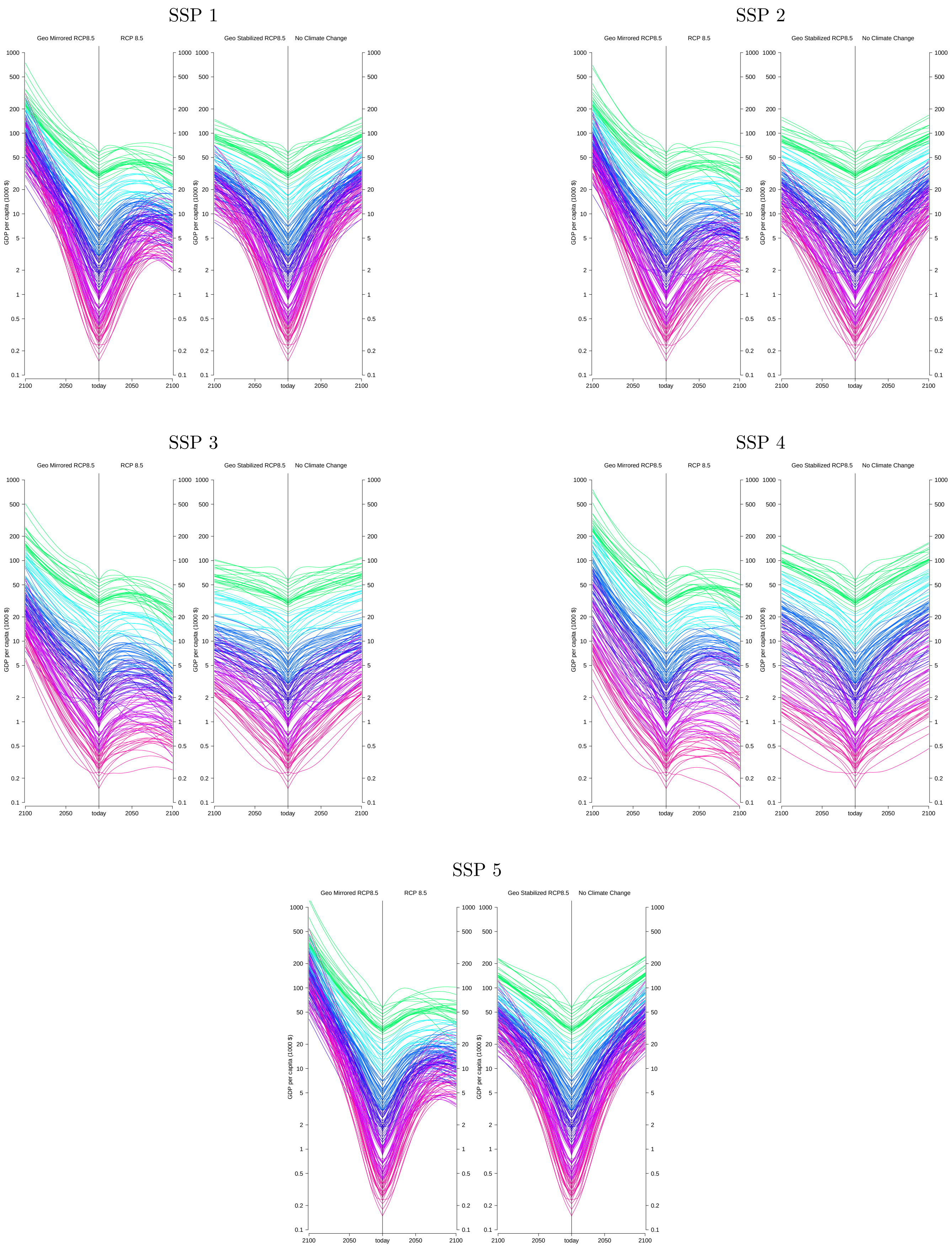
SSP 1
SSP 2
SSP 3
SSP 4
SSP 5
Geo Mirrored RCP8.5
RCP 8.5
Geo Stabilized RCP8.5
No Climate Change
GDP per capita (1000 $)
1000
500
200
100
50
20
10
5
2
1
0.5
0.2
0.1
2100
2050
today
2050
2100

d.

SSP 1

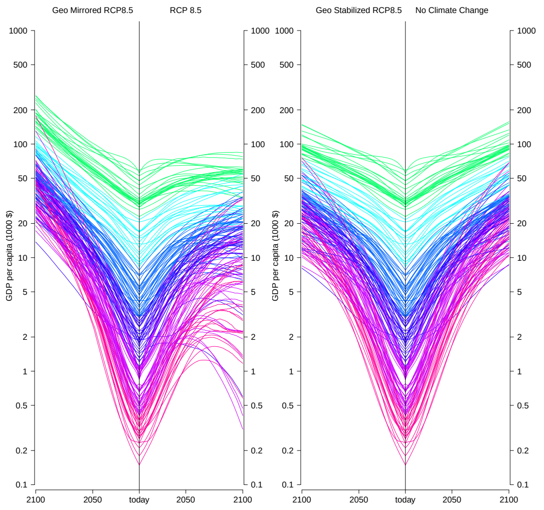

SSP 2

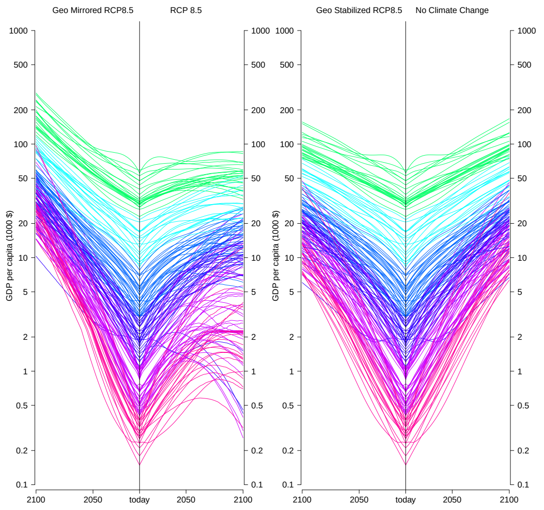

SSP 3

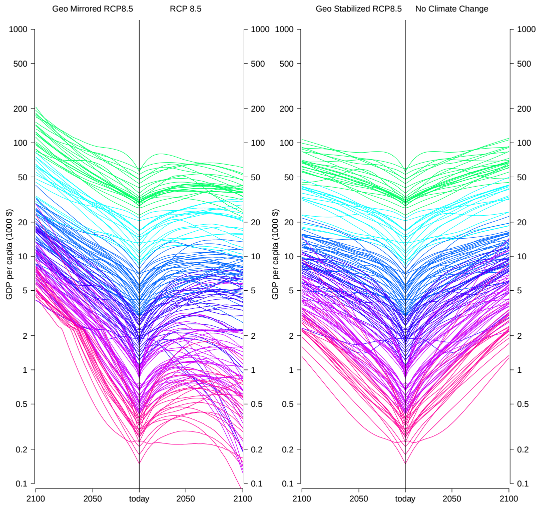

SSP 4

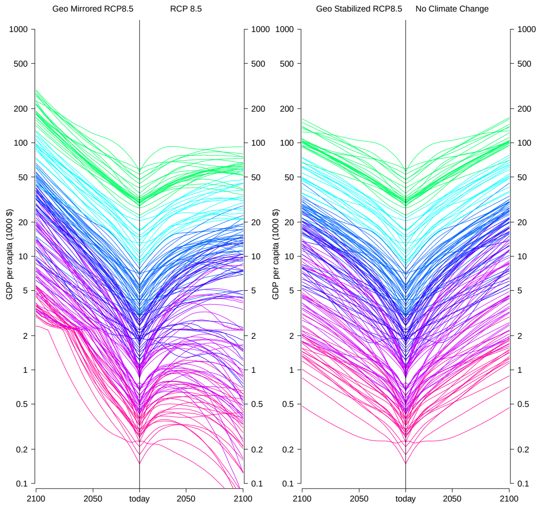

SSP 5

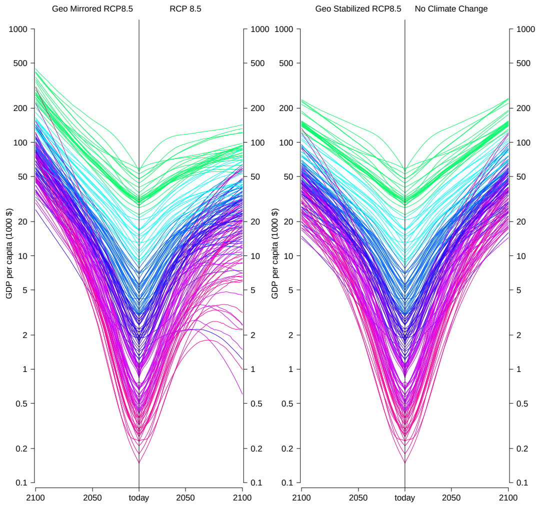

e.

SSP 1

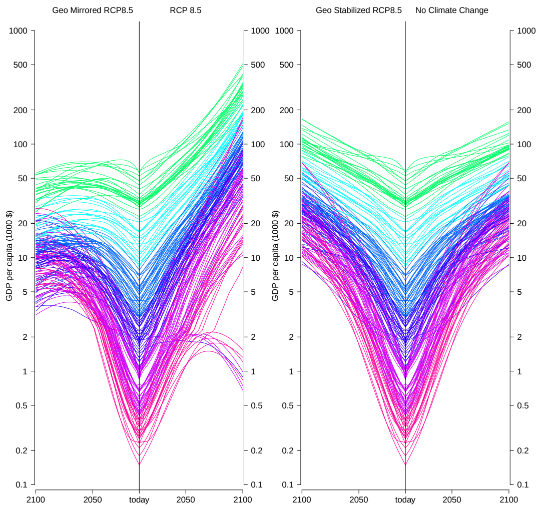

SSP 2

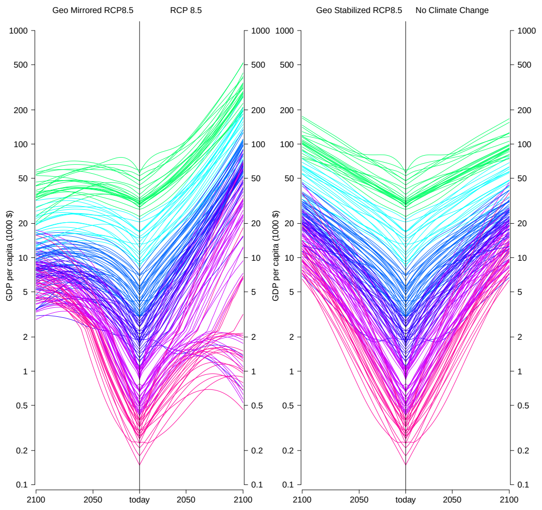

SSP 3

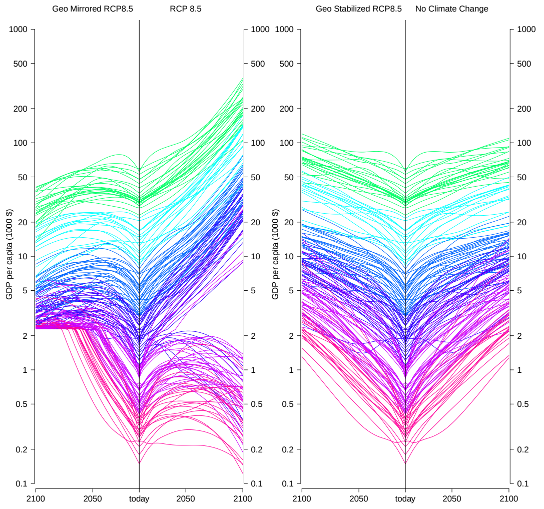

SSP 4

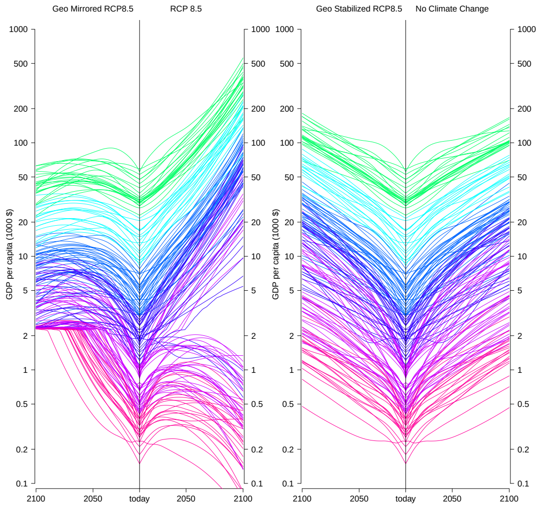

SSP 5

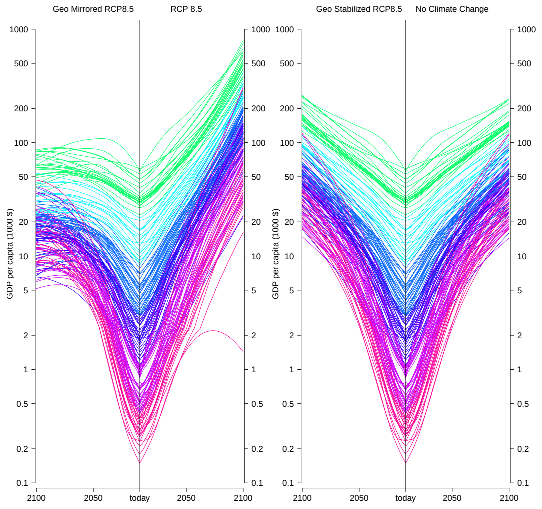

f.

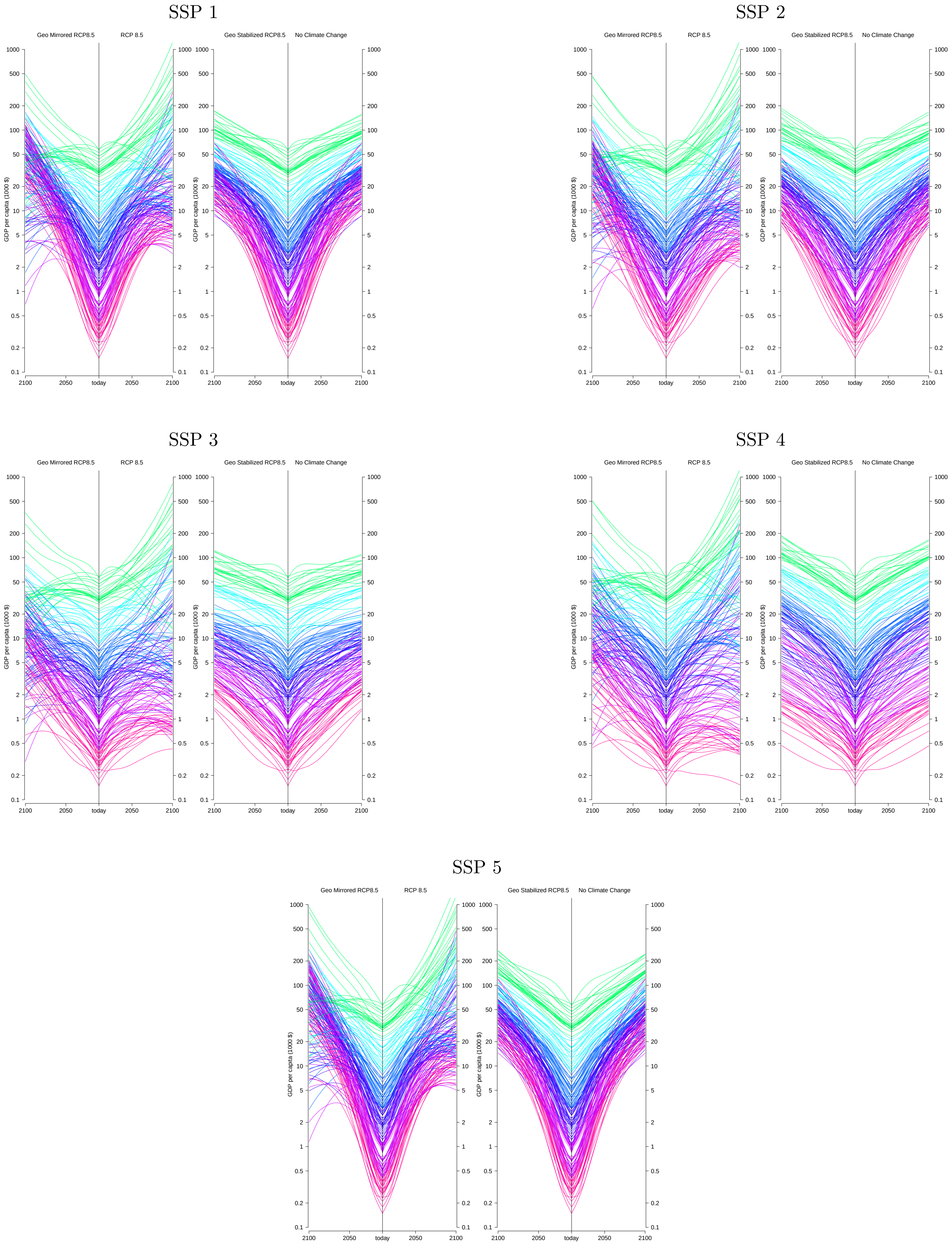
SSP 1
SSP 2
SSP 3
SSP 4
SSP 5
Geo Mirrored RCP8.5
RCP 8.5
Geo Stabilized RCP8.5
No Climate Change
GDP per capita (1000 $)
1000
500
200
100
50
20
10
5
2
1
0.5
0.2
0.1
2100
2050
today
2050
2100

g.

SSP 1

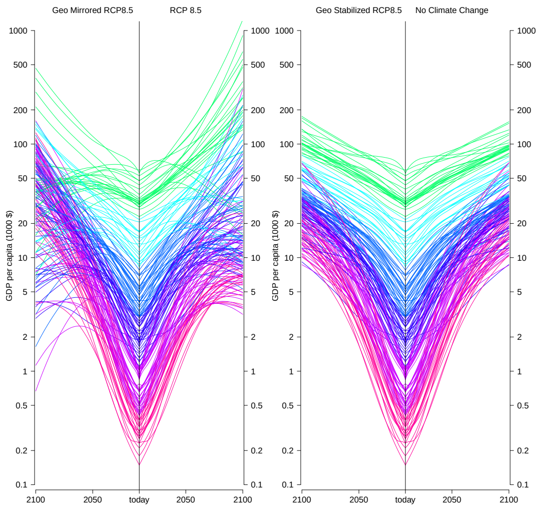

SSP 2

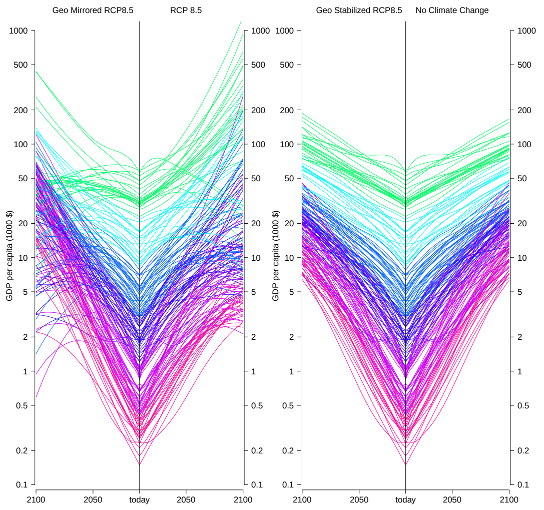

SSP 3

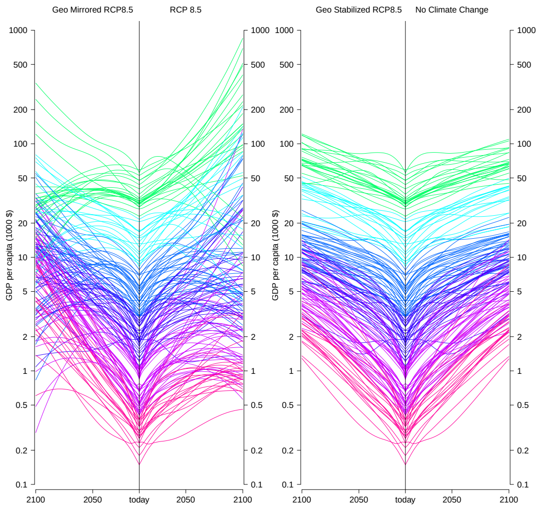

SSP 4

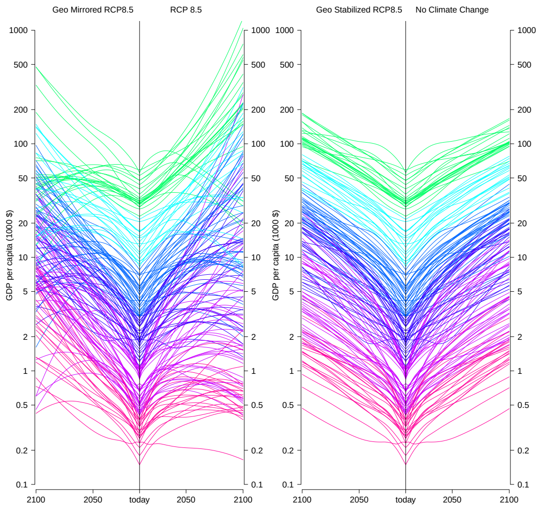

SSP 5

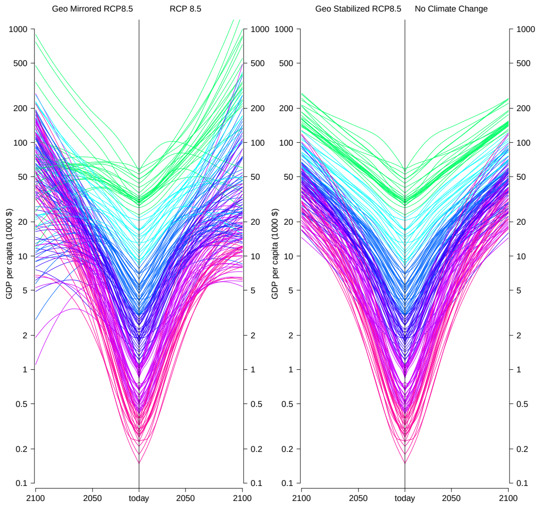

h.

SSP 1

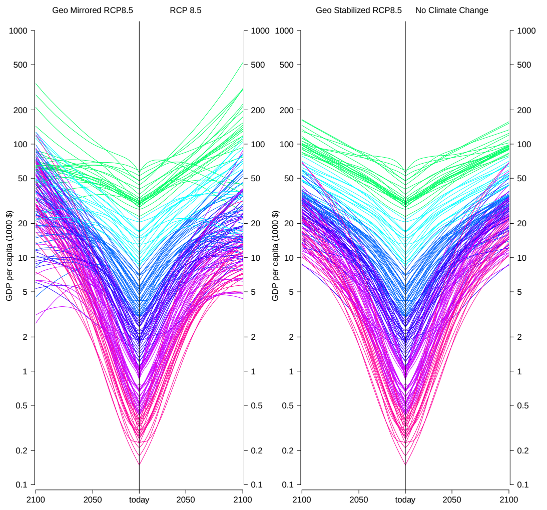

SSP 2

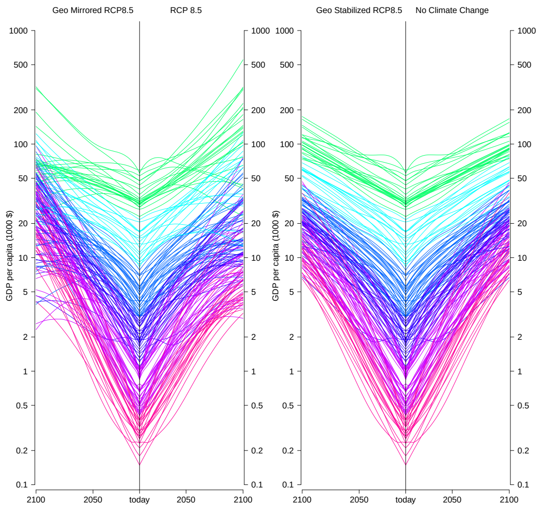

SSP 3

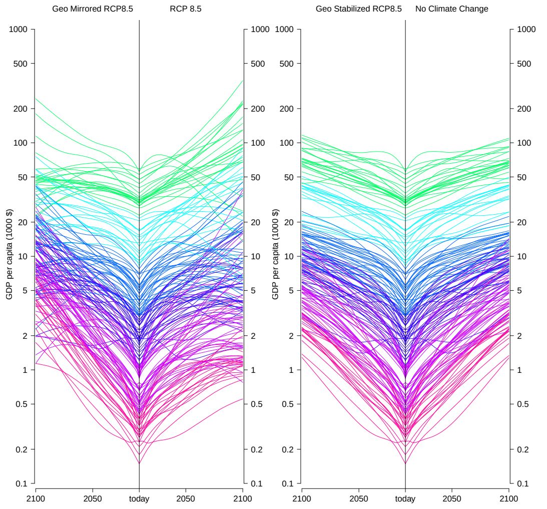

SSP 4

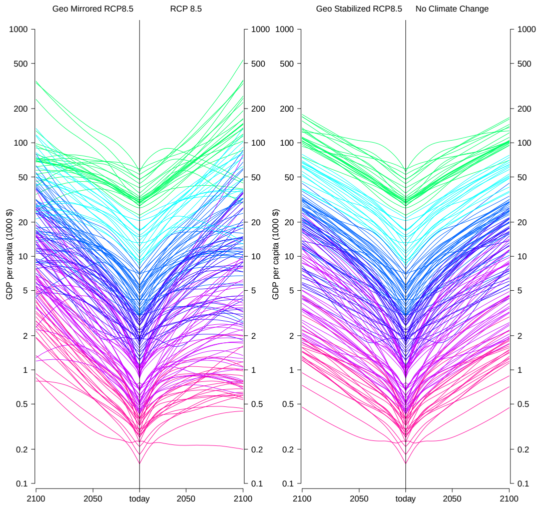

SSP 5

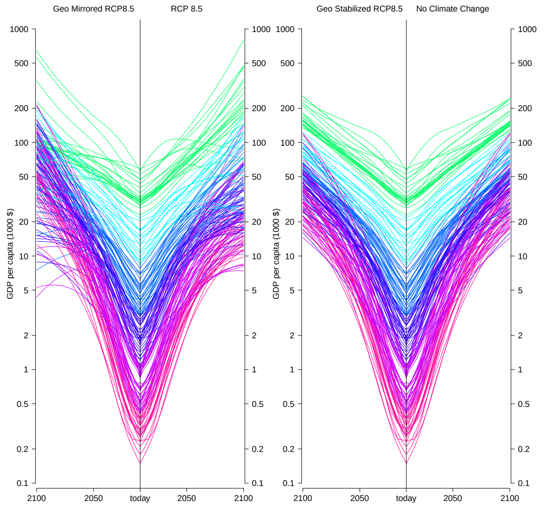

i.

SSP 1

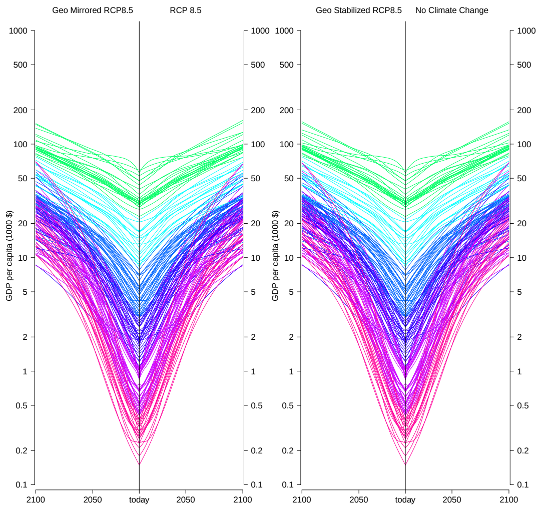

SSP 2

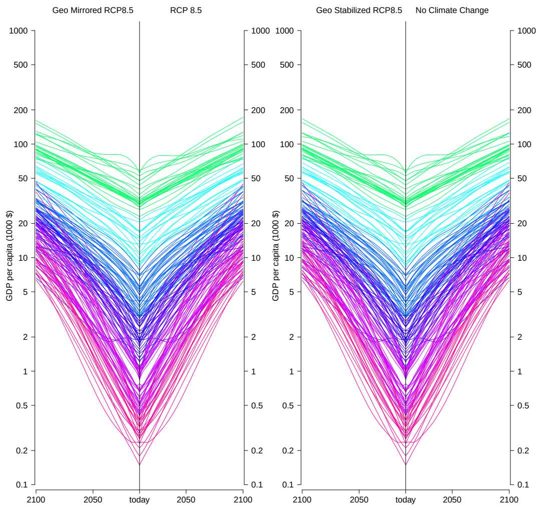

SSP 3

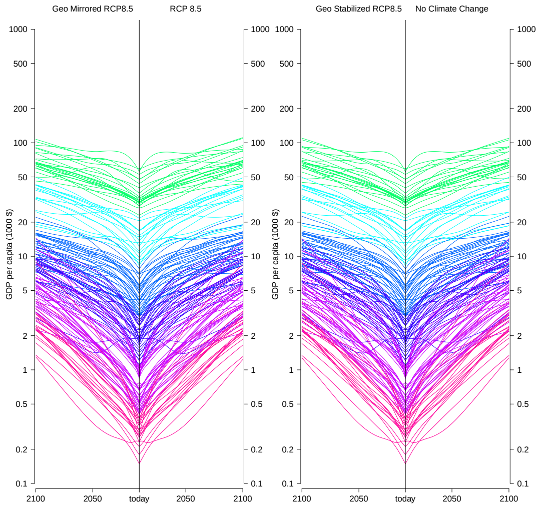

SSP 4

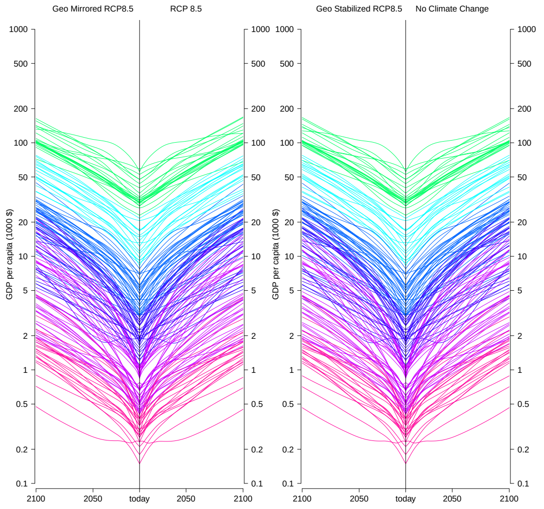

SSP 5

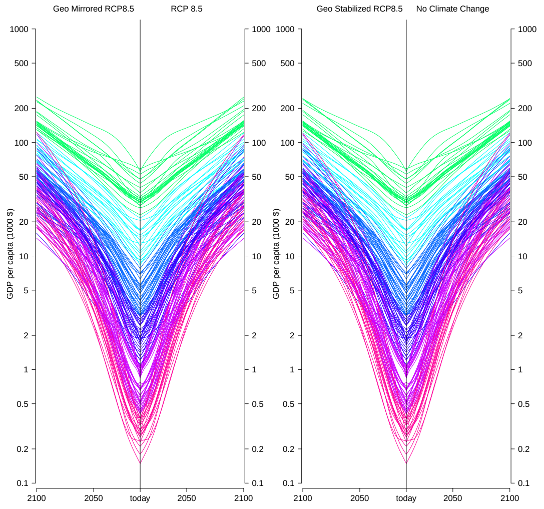

j.

SSP 1

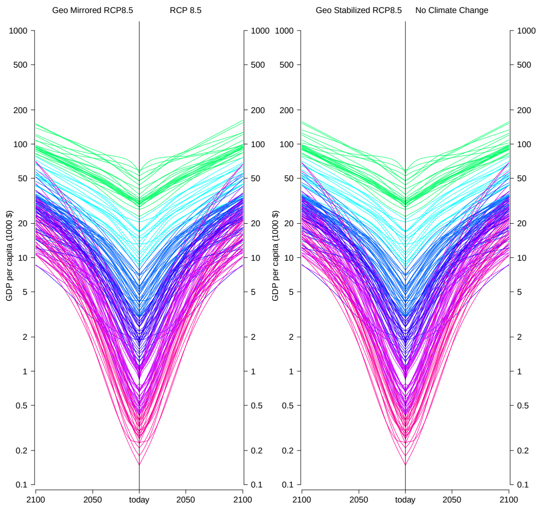

SSP 2

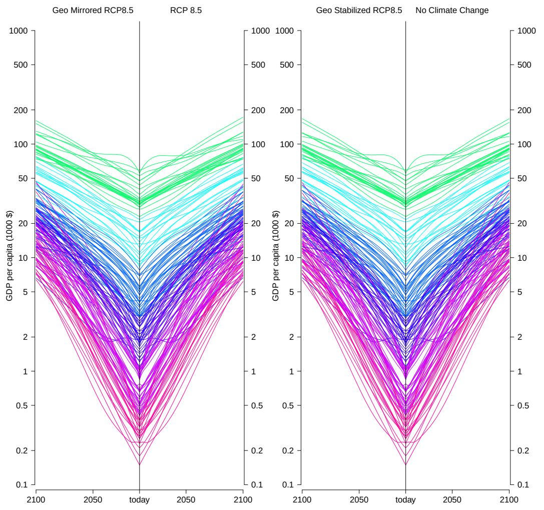

SSP 3

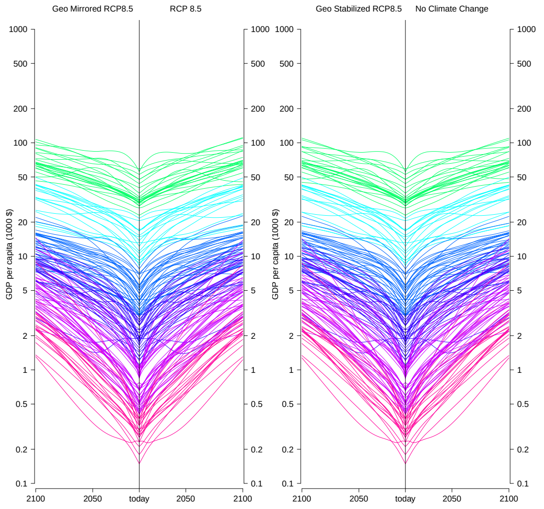

SSP 4

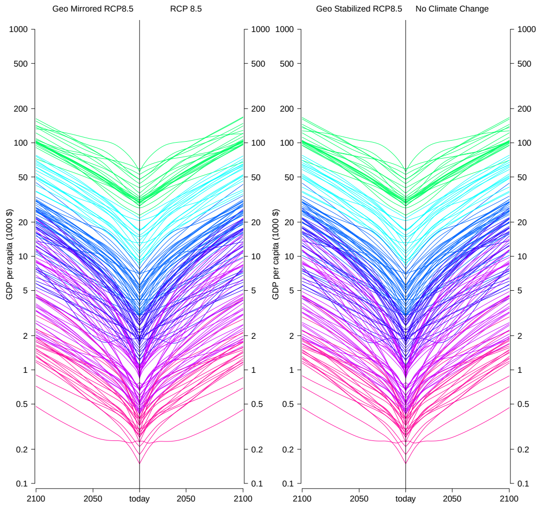

SSP 5

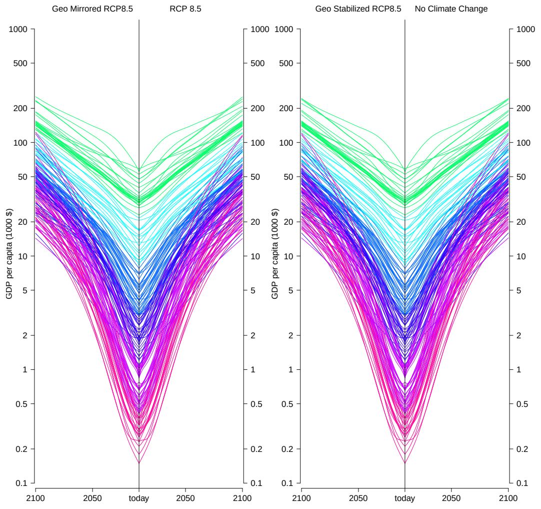

k.

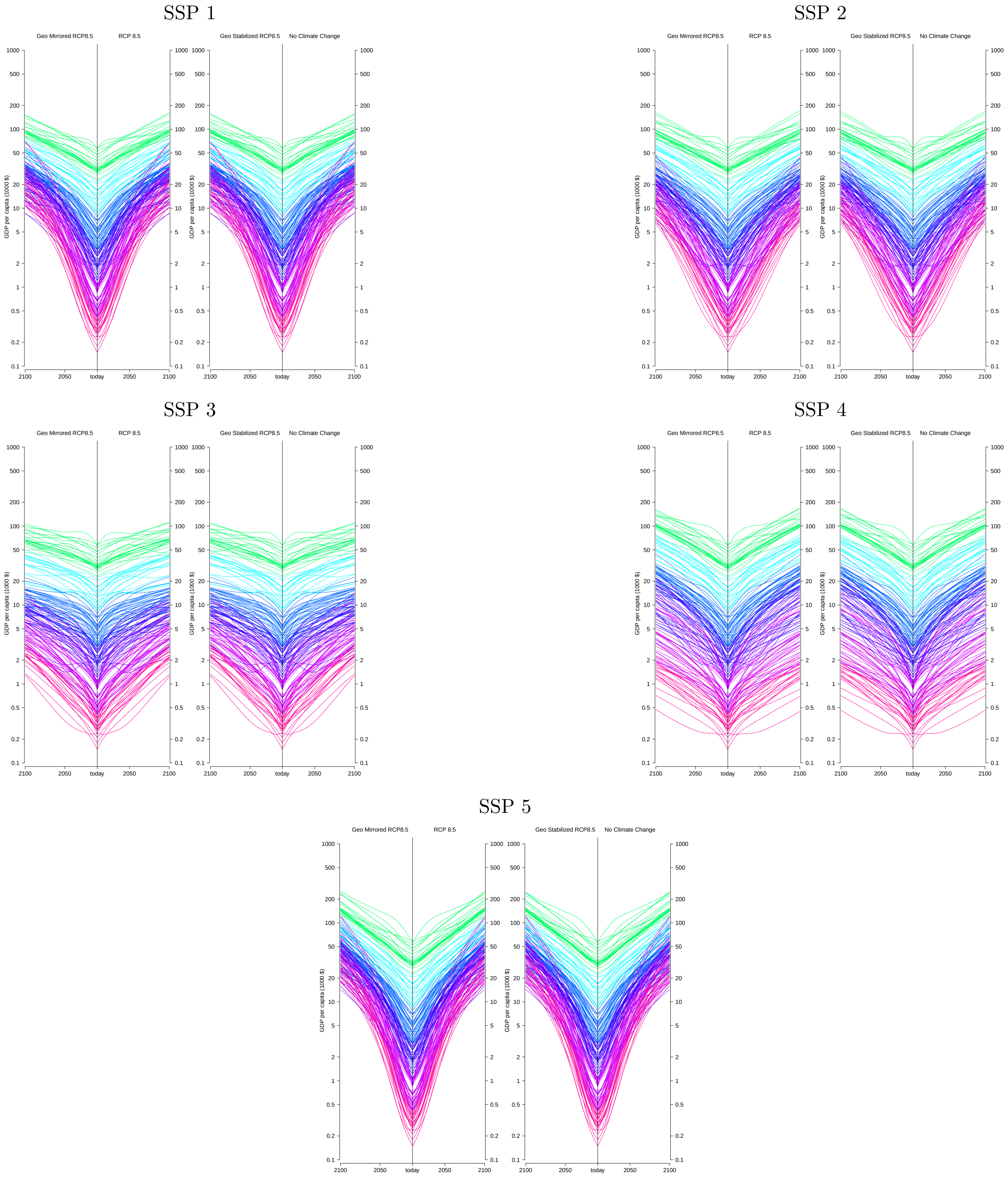

Supplementary Figure 6 | Country GDP per capita over time. Each line represents a single country. The color of each line represents the country's initial GDP per capita in 2010. Each panel shows a different SSP. Each subfigure shows a different climate impacts model. **a** uses the model from column (1) in Table S1; **b** uses column (2); **c** uses column (3); **d** uses column (4); **e** uses column (5); **f** uses column (6); **g** uses column (7); **h** uses column (8); **i** uses column (9); **j** uses column (10); **k** uses column (11).

a.

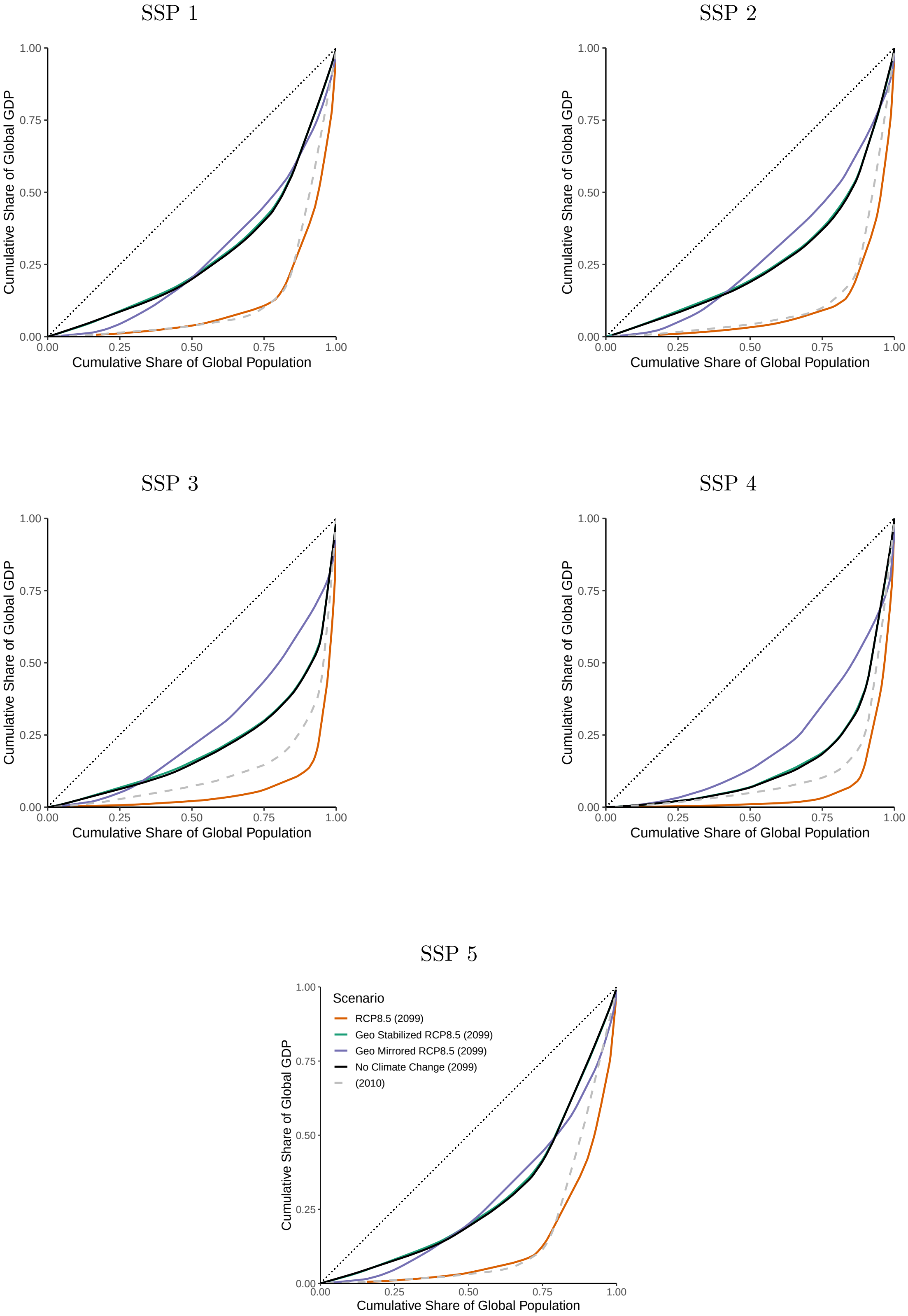
SSP 1
SSP 2
SSP 3
SSP 4
SSP 5
Cumulative Share of Global GDP
Cumulative Share of Global Population
0.00
0.25
0.50
0.75
1.00
Scenario
RCP8.5 (2099)
Geo Stabilized RCP8.5 (2099)
Geo Mirrored RCP8.5 (2099)
No Climate Change (2099)
(2010)

b.

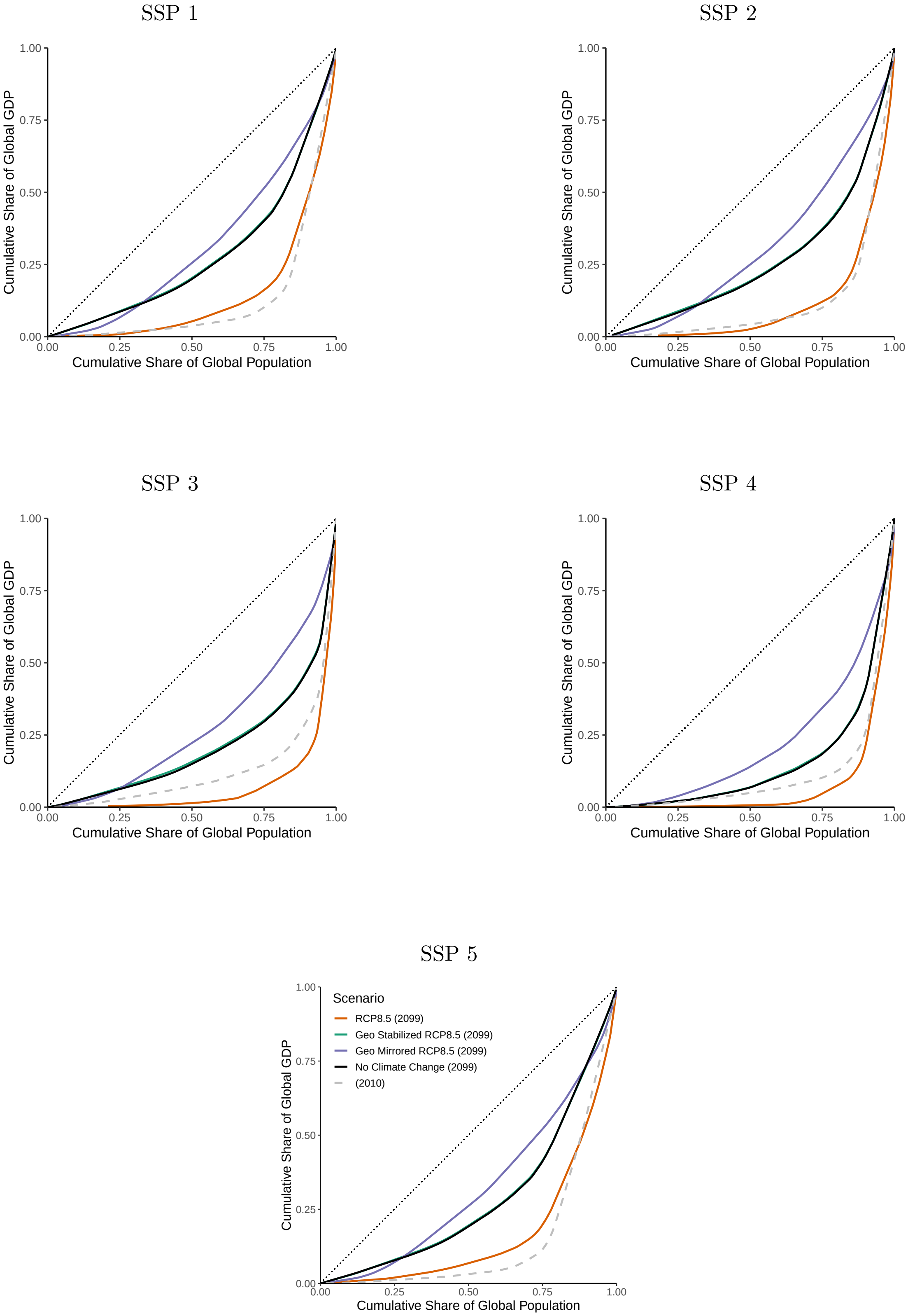
SSP 1
SSP 2
SSP 3
SSP 4
SSP 5
Cumulative Share of Global GDP
Cumulative Share of Global Population
0.00
0.25
0.50
0.75
1.00
Scenario
RCP8.5 (2099)
Geo Stabilized RCP8.5 (2099)
Geo Mirrored RCP8.5 (2099)
No Climate Change (2099)
(2010)

c.

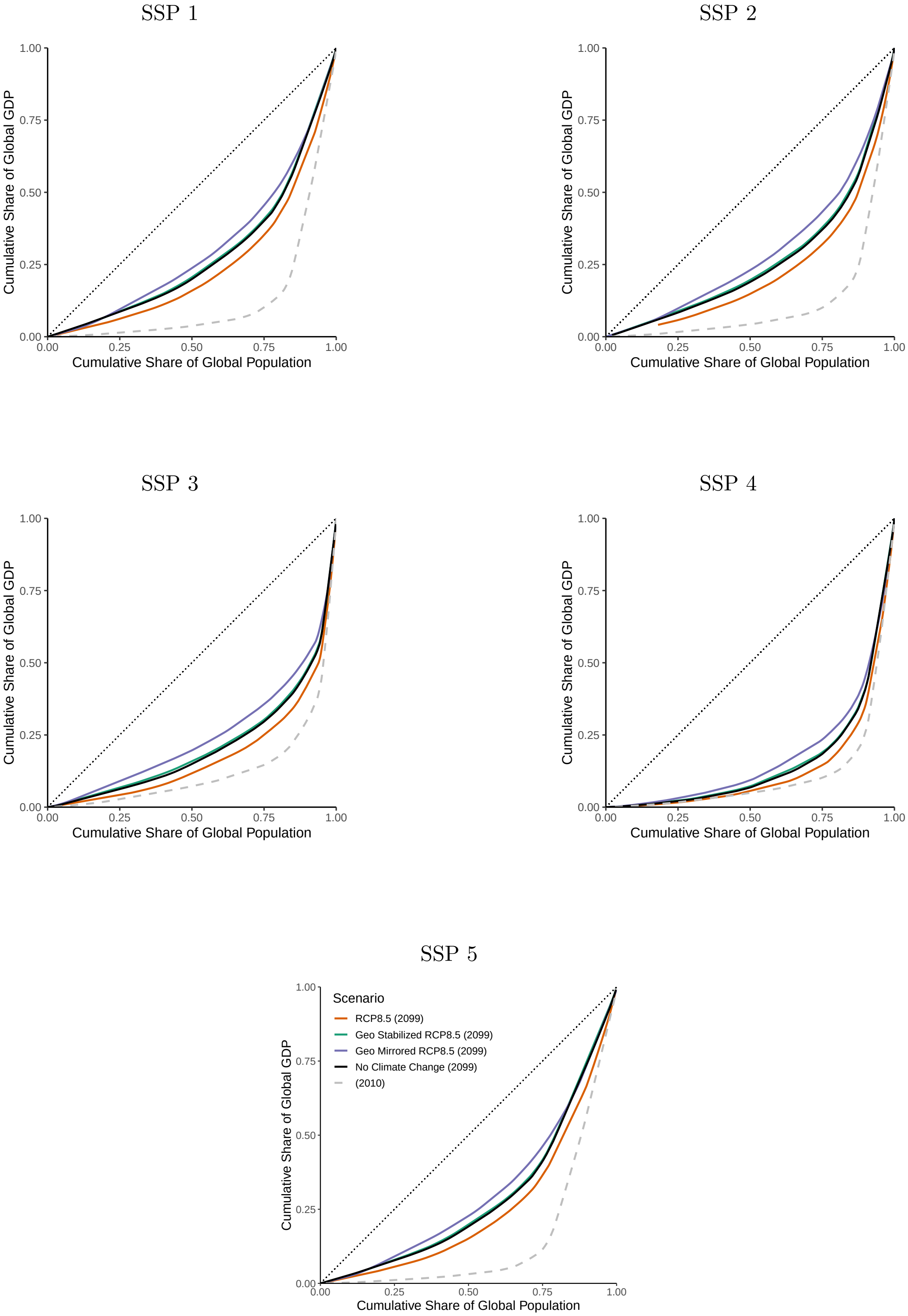

SSP 1
SSP 2
SSP 3
SSP 4
SSP 5
Cumulative Share of Global GDP
Cumulative Share of Global Population
0.00
0.25
0.50
0.75
1.00
Scenario
RCP8.5 (2099)
Geo Stabilized RCP8.5 (2099)
Geo Mirrored RCP8.5 (2099)
No Climate Change (2099)
(2010)

d.

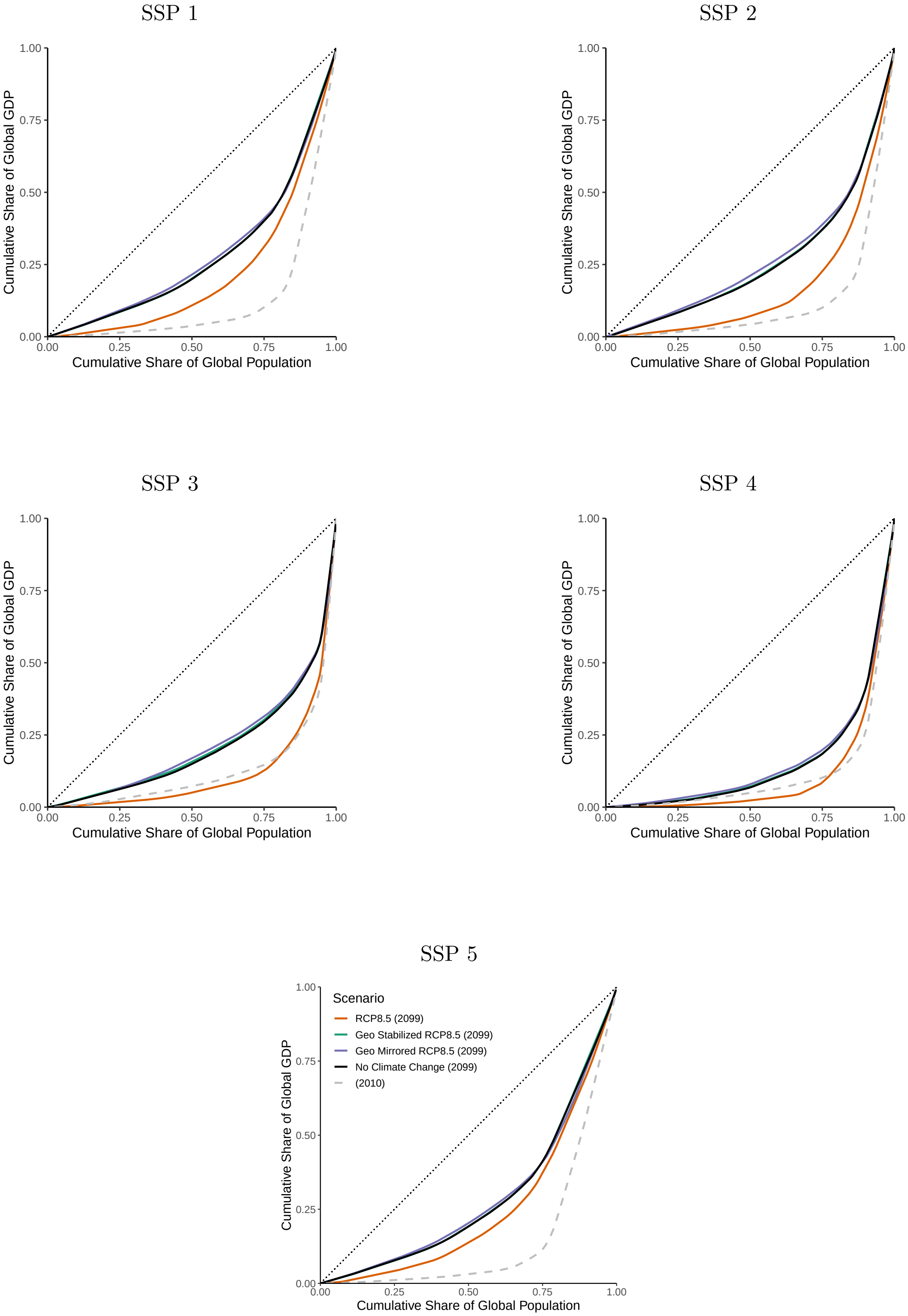
SSP 1
SSP 2
SSP 3
SSP 4
SSP 5
Cumulative Share of Global GDP
Cumulative Share of Global Population
0.00
0.25
0.50
0.75
1.00
Scenario
RCP8.5 (2099)
Geo Stabilized RCP8.5 (2099)
Geo Mirrored RCP8.5 (2099)
No Climate Change (2099)
(2010)

e.

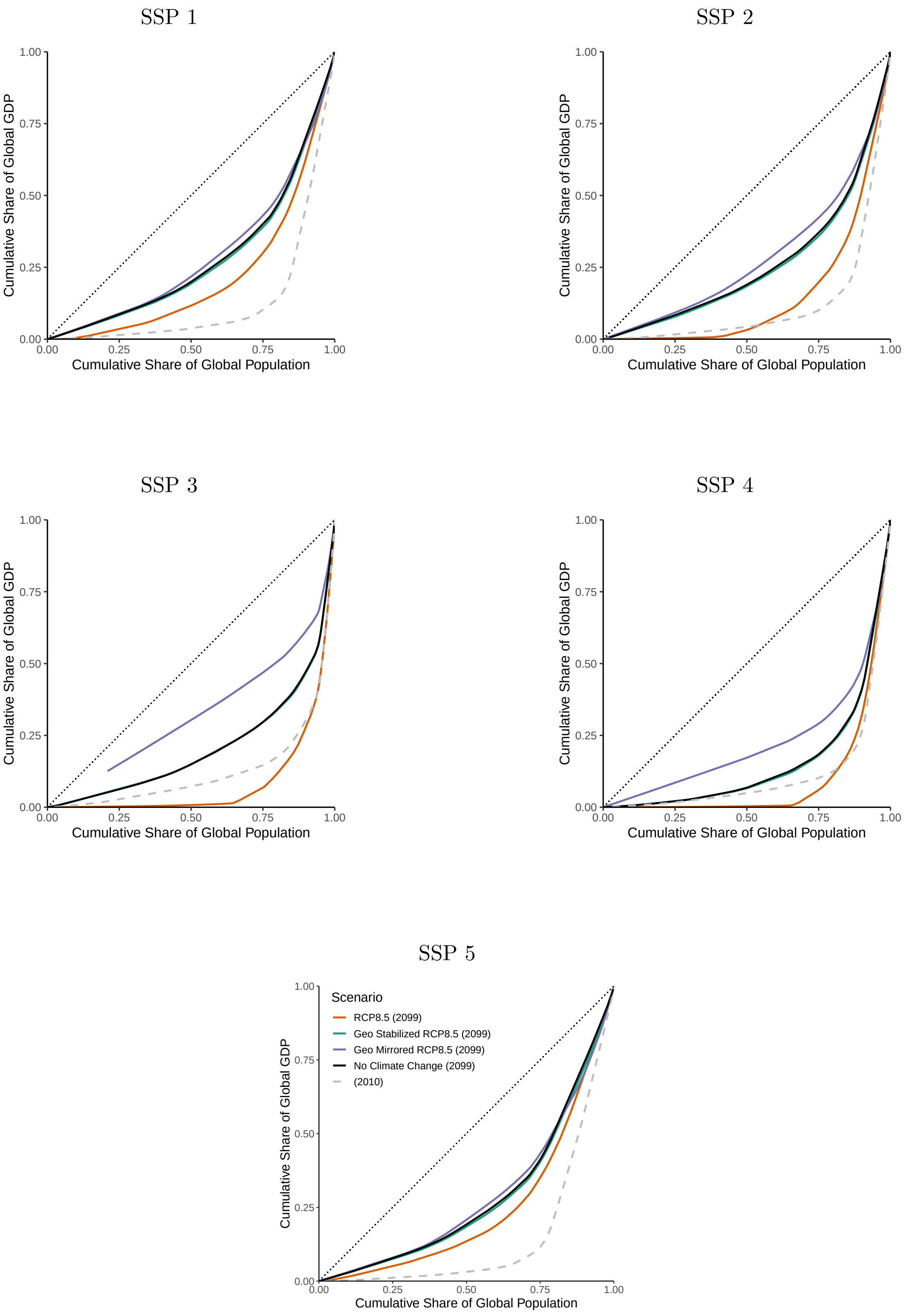
SSP 1
SSP 2
SSP 3
SSP 4
SSP 5
Cumulative Share of Global GDP
Cumulative Share of Global Population
0.00
0.25
0.50
0.75
1.00
Scenario
RCP8.5 (2099)
Geo Stabilized RCP8.5 (2099)
Geo Mirrored RCP8.5 (2099)
No Climate Change (2099)
(2010)

f.

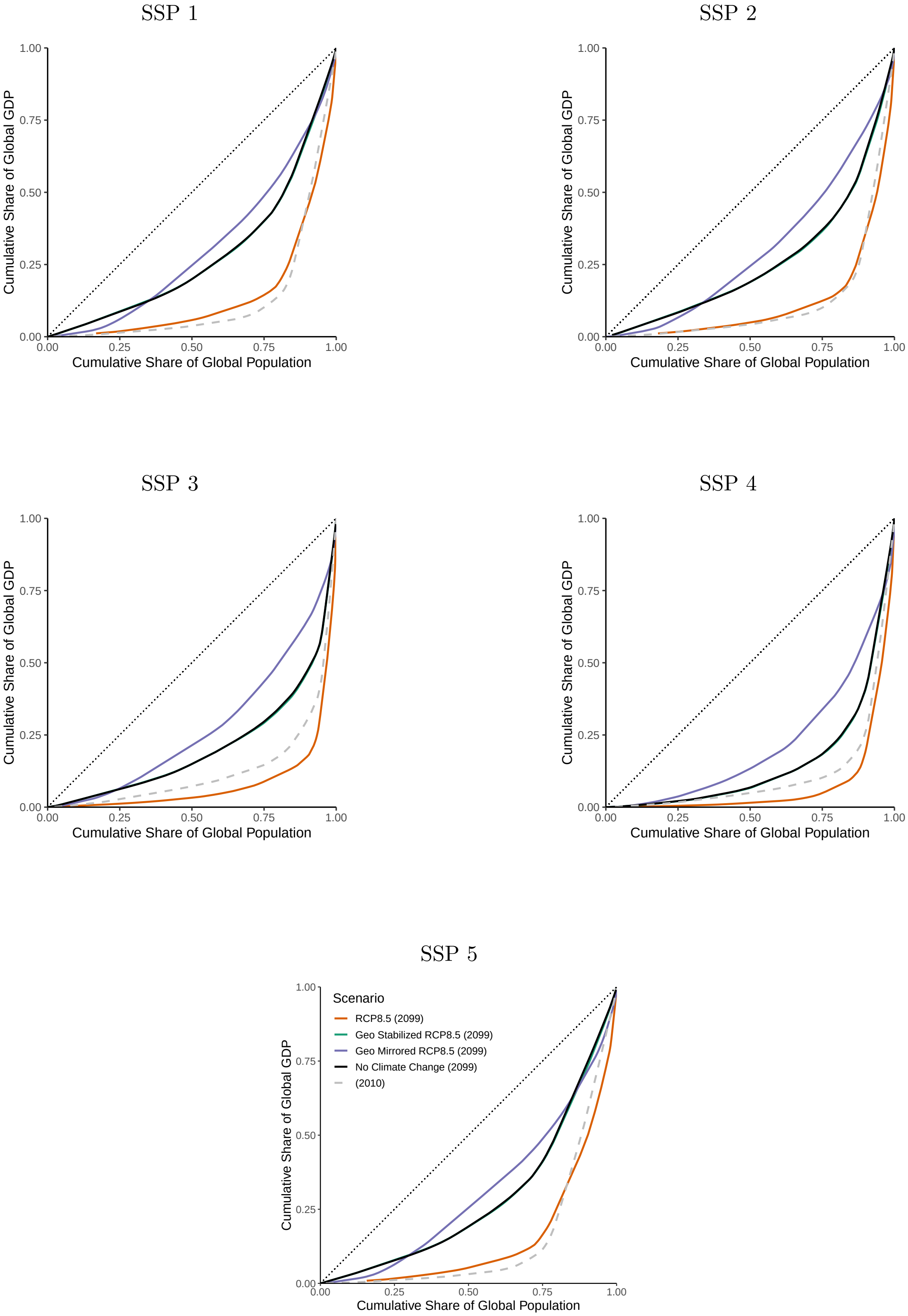
SSP 1
SSP 2
SSP 3
SSP 4
SSP 5
Cumulative Share of Global GDP
Cumulative Share of Global Population
0.00
0.25
0.50
0.75
1.00
Scenario
RCP8.5 (2099)
Geo Stabilized RCP8.5 (2099)
Geo Mirrored RCP8.5 (2099)
No Climate Change (2099)
(2010)

g.

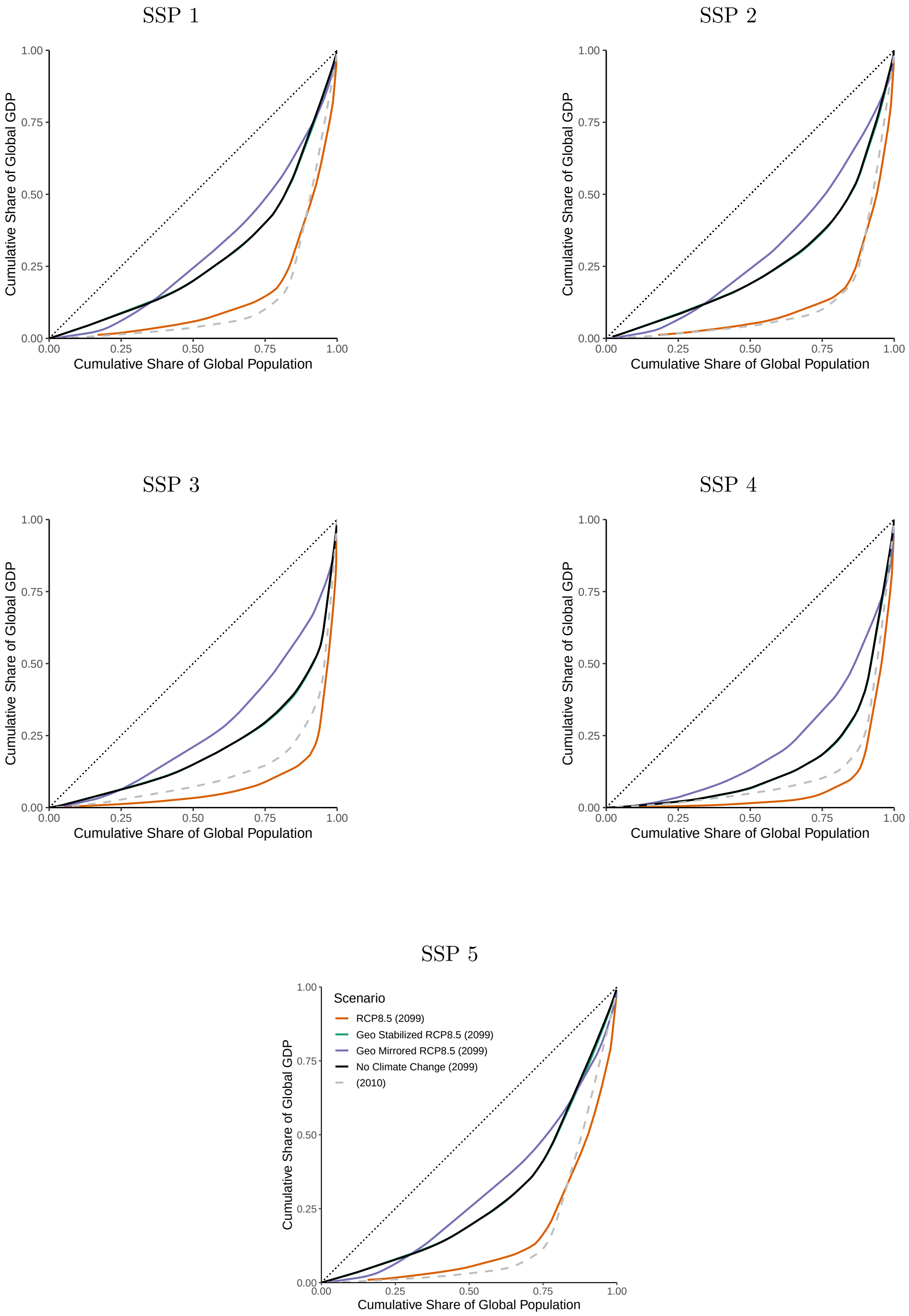
SSP 1
SSP 2
SSP 3
SSP 4
SSP 5
Cumulative Share of Global GDP
Cumulative Share of Global Population
0.00
0.25
0.50
0.75
1.00
Scenario
RCP8.5 (2099)
Geo Stabilized RCP8.5 (2099)
Geo Mirrored RCP8.5 (2099)
No Climate Change (2099)
(2010)

h.

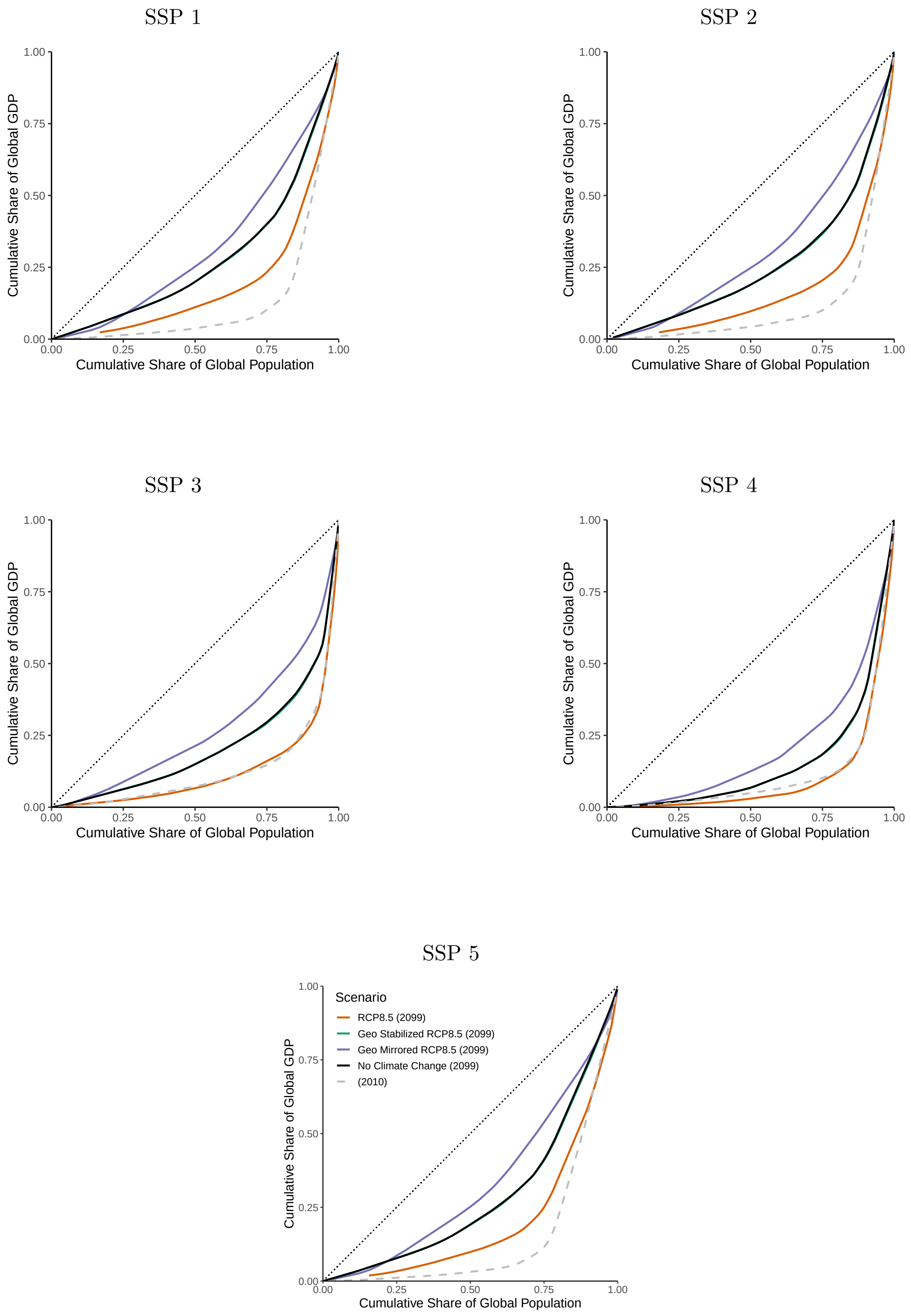
SSP 1
SSP 2
SSP 3
SSP 4
SSP 5
Cumulative Share of Global GDP
Cumulative Share of Global Population
0.00
0.25
0.50
0.75
1.00
Scenario
RCP8.5 (2099)
Geo Stabilized RCP8.5 (2099)
Geo Mirrored RCP8.5 (2099)
No Climate Change (2099)
(2010)

i.

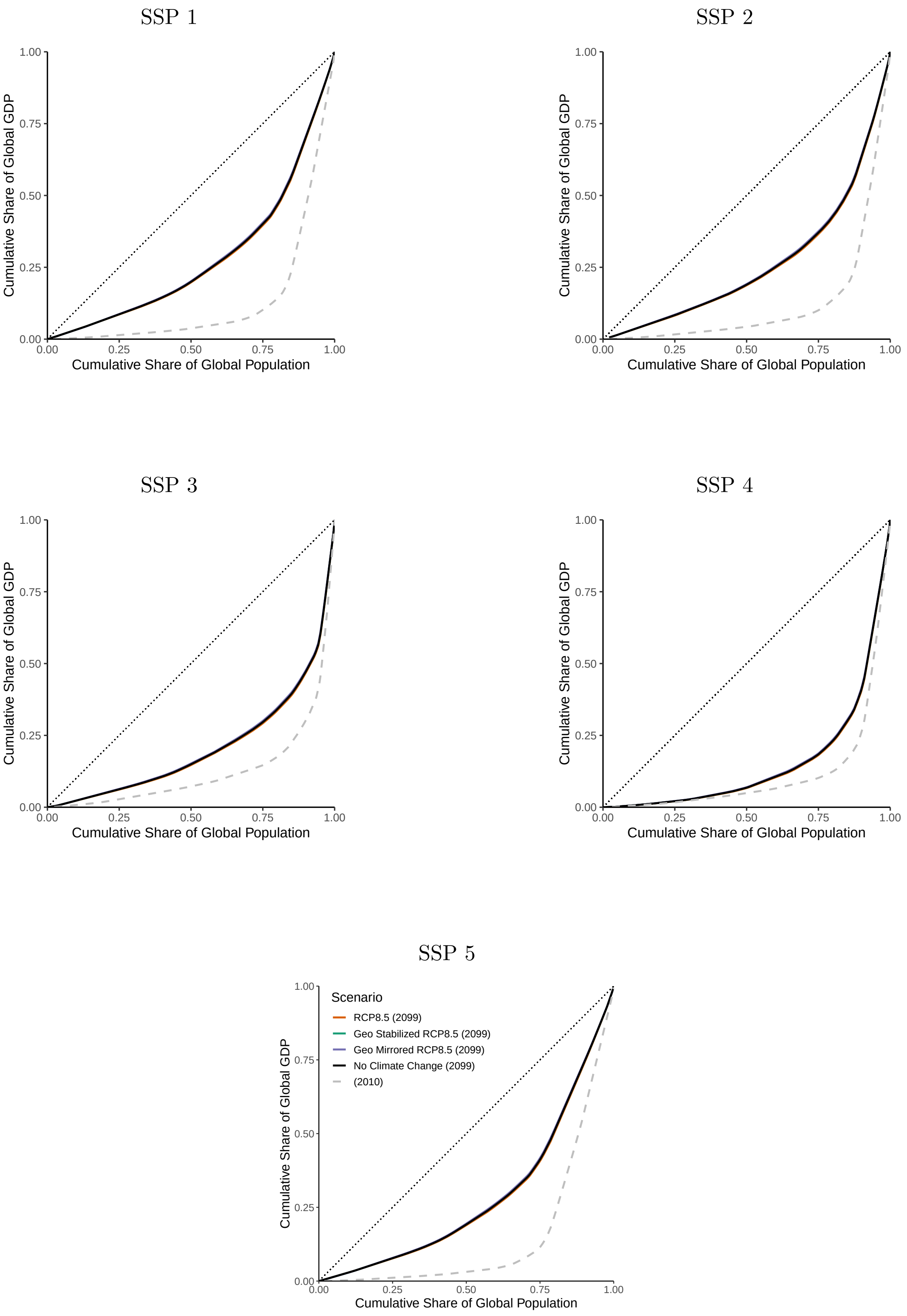
SSP 1
SSP 2
SSP 3
SSP 4
SSP 5
Cumulative Share of Global GDP
Cumulative Share of Global Population
0.00
0.25
0.50
0.75
1.00
Scenario
RCP8.5 (2099)
Geo Stabilized RCP8.5 (2099)
Geo Mirrored RCP8.5 (2099)
No Climate Change (2099)
(2010)

j.

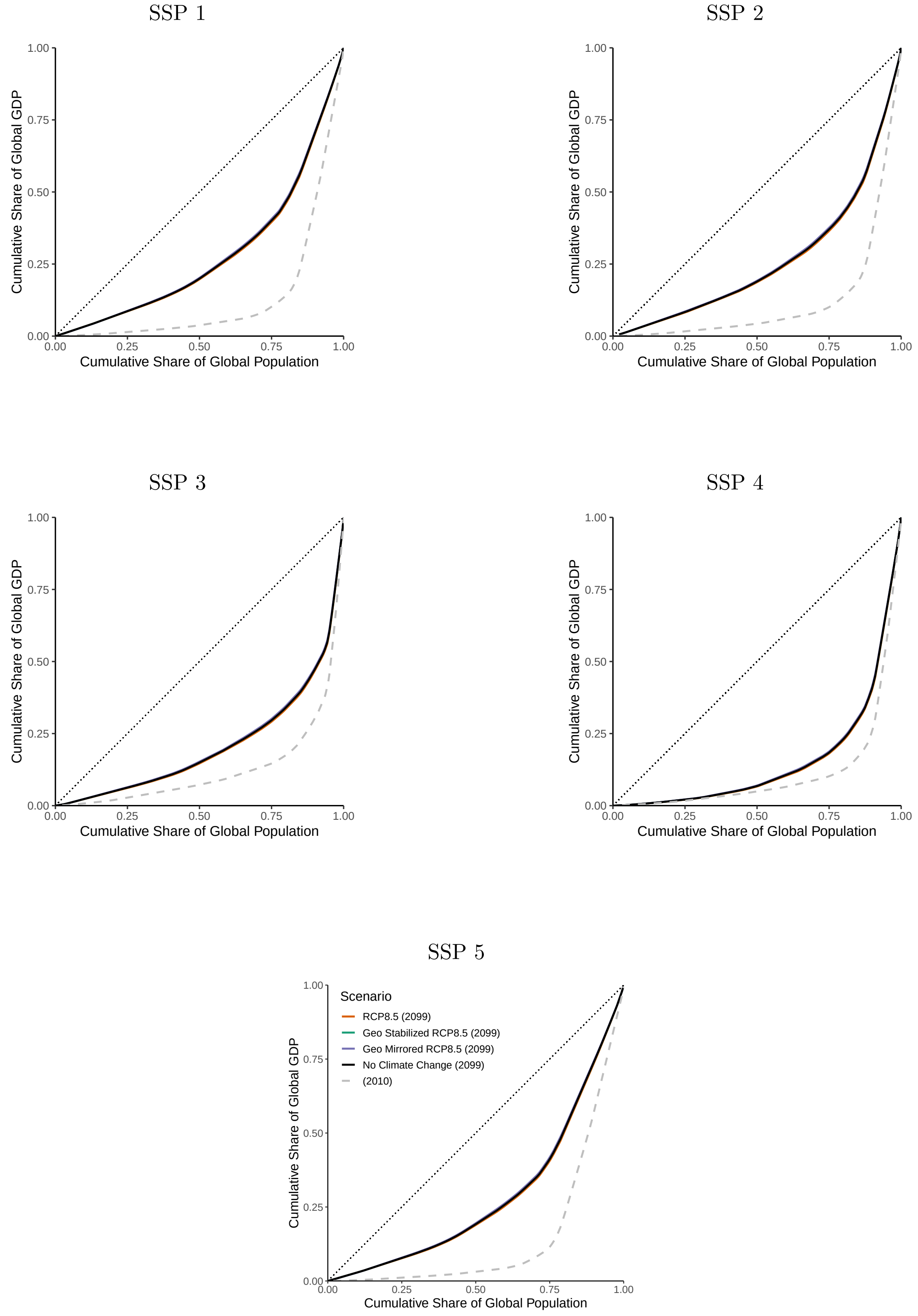
SSP 1
SSP 2
SSP 3
SSP 4
SSP 5
Cumulative Share of Global GDP
Cumulative Share of Global Population
0.00
0.25
0.50
0.75
1.00
Scenario
RCP8.5 (2099)
Geo Stabilized RCP8.5 (2099)
Geo Mirrored RCP8.5 (2099)
No Climate Change (2099)
(2010)

k.

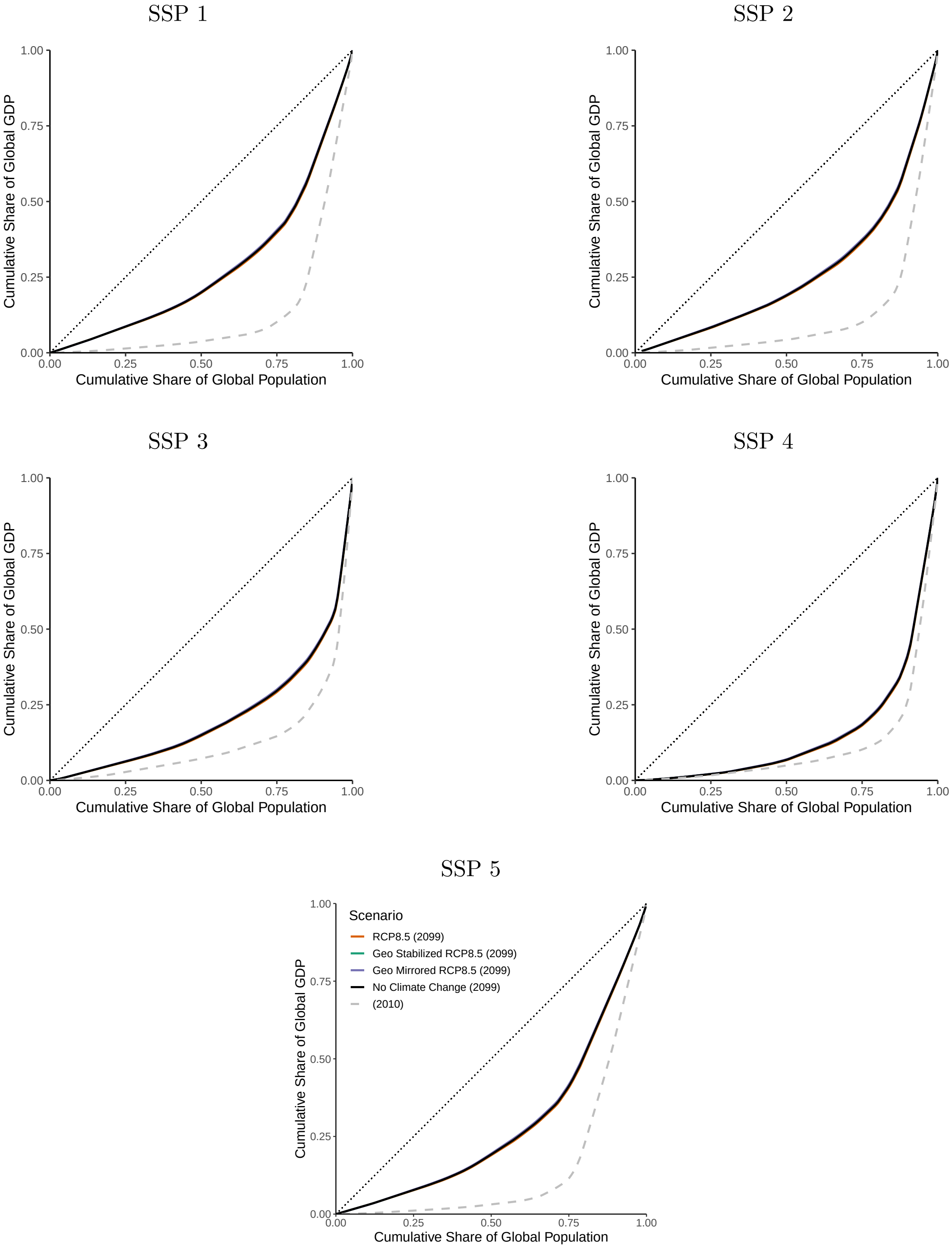

Supplementary Figure 7 | Lorenz Curve for share of global GDP. Each line represents the distribution of the projected share of global GDP in 2099 for a different climate scenario in addition to the initial distribution in 2010. Each panel displays a different SSP. Each subfigure displays a different climate impacts model. **a** uses the model from column (1) in Table S1; **b** uses column (2); **c** uses column (3); **d** uses column (4); **e** uses column (5); **f** uses column (6); **g** uses column (7); **h** uses column (8); **i** uses column (9); **j** uses column (10); **k** uses column (11).

a. Geo Stable SSP 1

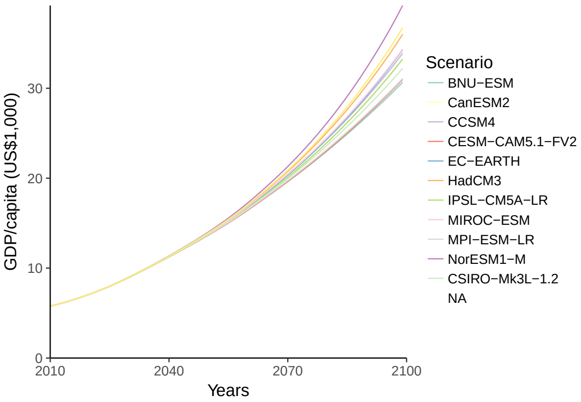

b. Geo Mirror SSP 1

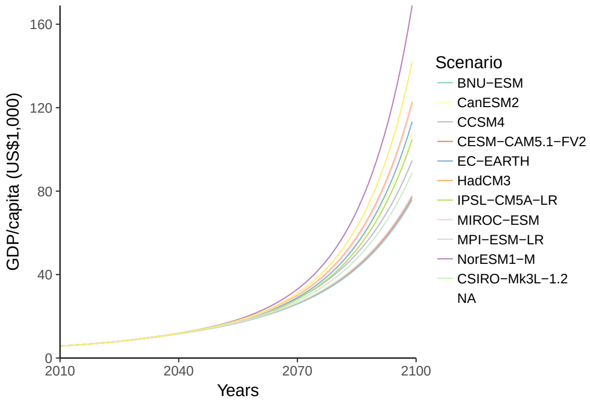

c. Geo Stable SSP 2

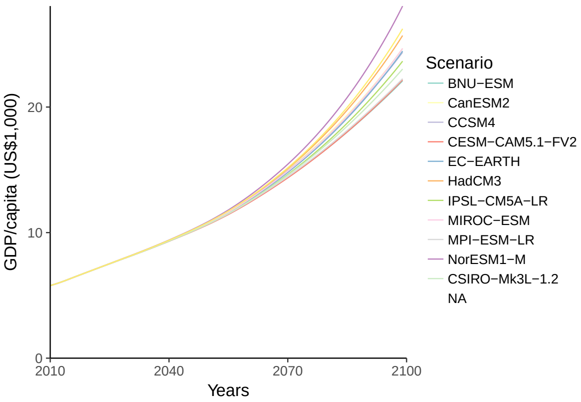

d. Geo Mirror SSP 2

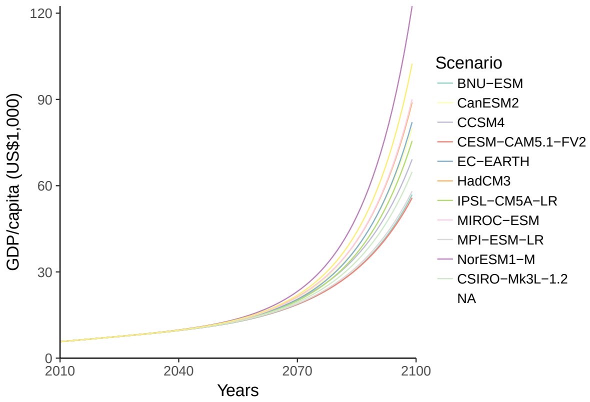

e. Geo Stable SSP 3

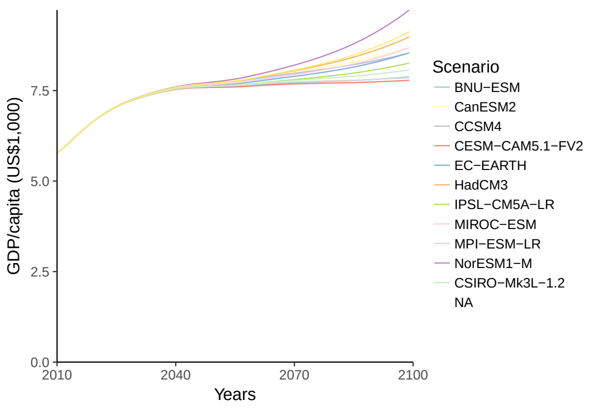

f. Geo Mirror SSP 3

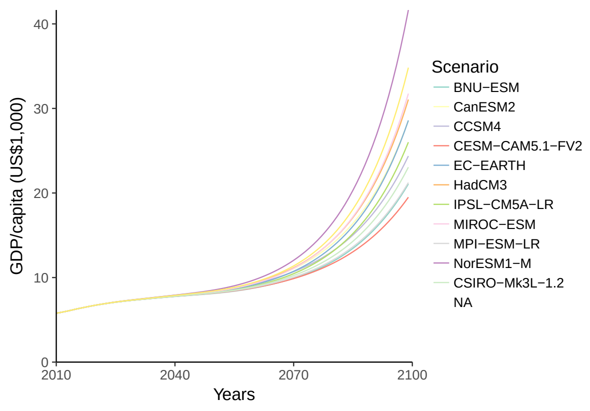

g. Geo Stable SSP 4

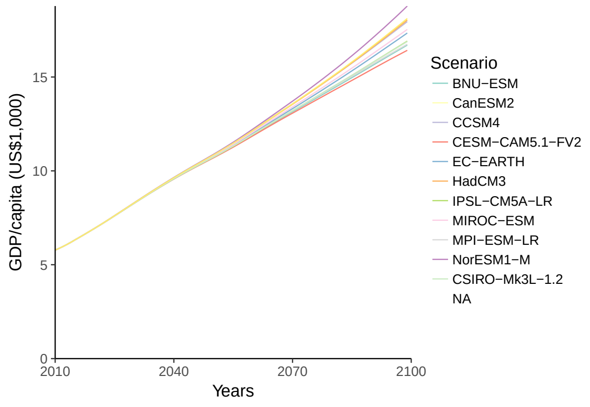

h. Geo Mirror SSP 4

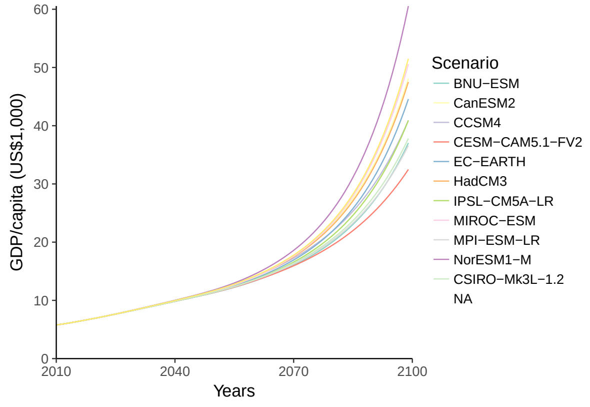

i. Geo Stable SSP 5 **j.** Geo Mirror SSP 5

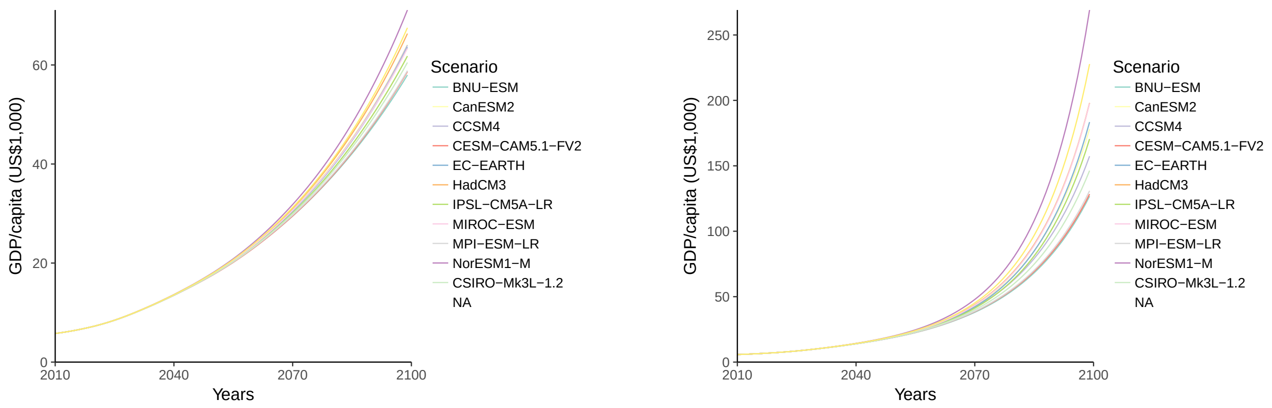

Supplementary Figure 8 | Projected global GDP per capita over the 21st Century across precipitation and temperature response to solar geoengineering. Economic projections for Geoengineering Stabilized (a) SSP 1, (c) SSP2, (e) SSP3, (g) SSP4, (i) SSP5 and Geoengineering Mirrored (b) SSP1, (d) SSP2, (f) SSP3, (h) SSP4, (j) SSP5. Each line represents the median economic projection for temperature and precipitation response for each GeoMIP climate model individually. Results are for the model in column 1 of Supplementary Table S1.

a. Geo Stable SSP 1

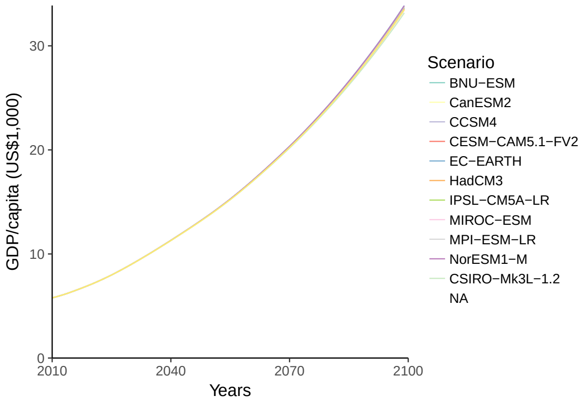

b. Geo Mirror SSP 1

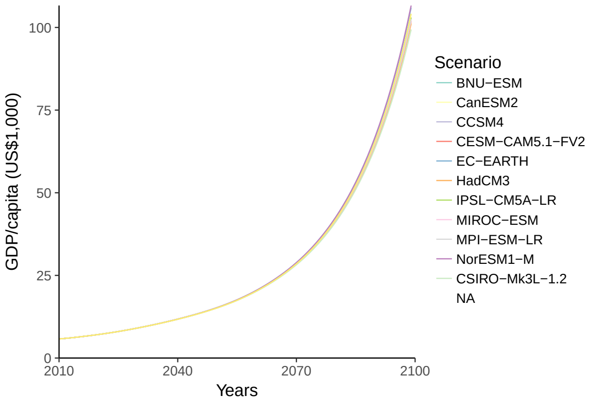

c. Geo Stable SSP 2

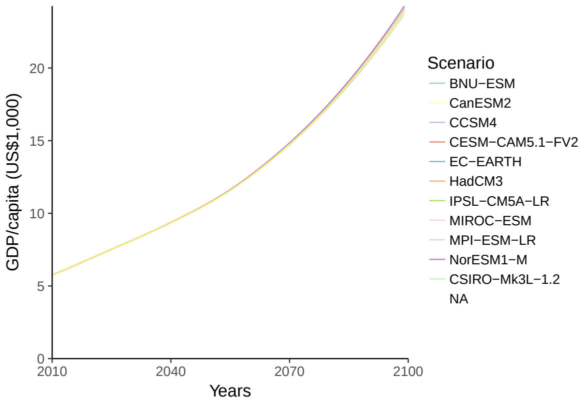

d. Geo Mirror SSP 2

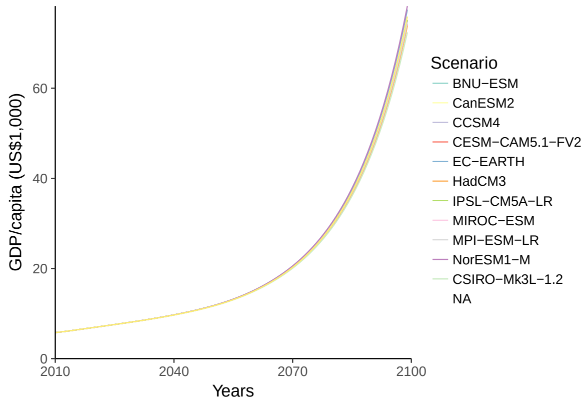

e. Geo Stable SSP 3

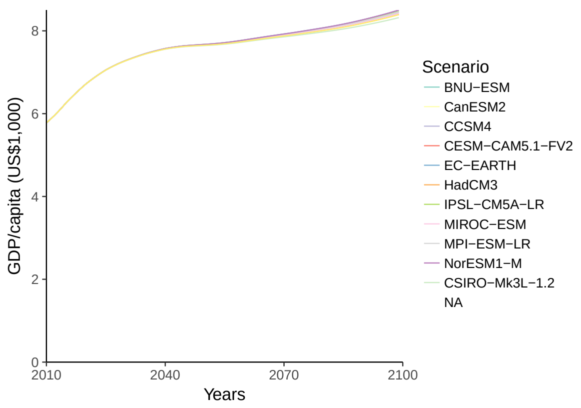

f. Geo Mirror SSP 3

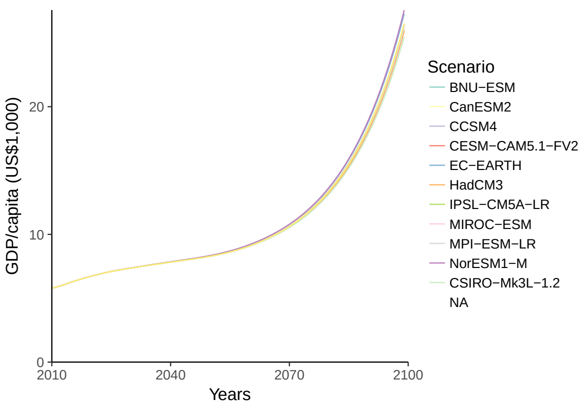

g. Geo Stable SSP 4

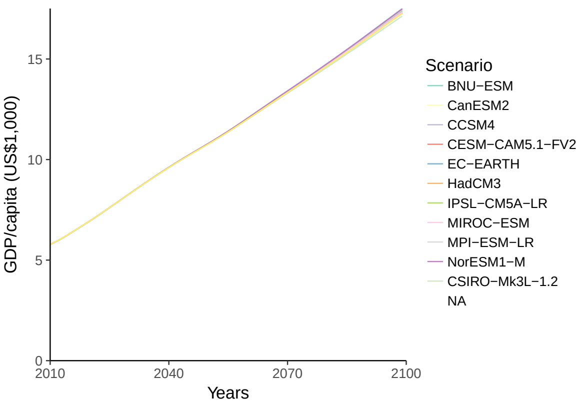

h. Geo Mirror SSP 4

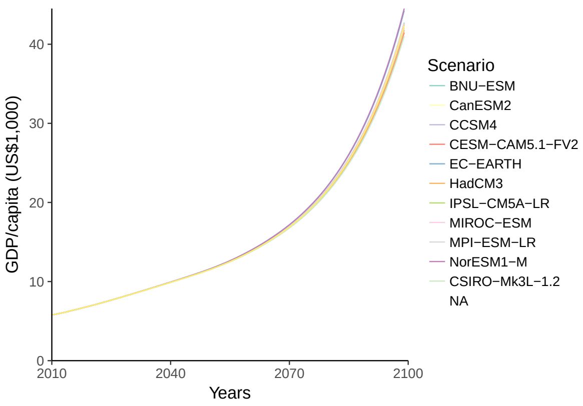

i. Geo Stable SSP 5 **j.** Geo Mirror SSP 5

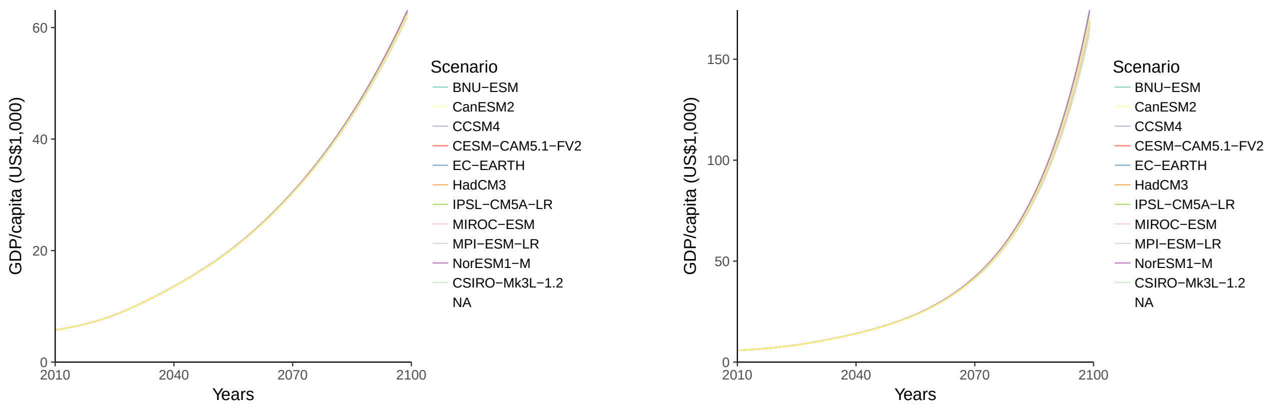

Supplementary Figure 9 | Projected global GDP per capita over the 21st Century across precipitation response to solar geoengineering. Economic projections for Geoengineering Stabilized (a) SSP 1, (c) SSP2, (e) SSP3, (g) SSP4, (i) SSP5 and Geoengineering Mirrored (b) SSP1, (d) SSP2, (f) SSP3, (h) SSP4, (j) SSP5. Each line represents the median economic projection for GeoMIP mean ensemble temperature response and precipitation response for each GeoMIP climate model individually. Results are for the model in column 1 of Supplementary Table S1.

Supplementary Tables

Supplementary Table 1 | Regression Results. Summary of regression results for the econometrically estimated historical climate-economy relationship. Estimated using fixed-effects models of 165 countries from 1960-2010.

	(1)	(2)	(3)	(4)	(5)	(6)	(7)	(8)	(9)	(10)	(11)
Temp	0.0127***	0.00890*	0.00978**	0.0104*	0.00606	0.0132***	0.0133***	0.0111***			
	(0.00325)	(0.00421)	(0.00355)	(0.00454)	(0.00350)	(0.00369)	(0.00370)	(0.00314)			
Temp Sq.	-0.000487***	-0.000316	-0.000466***	-0.000454**		-0.000385***	-0.000381***	-0.000442***			
	(0.000103)	(0.000162)	(0.000110)	(0.000172)		(0.000104)	(0.000105)	(0.000126)			
Precip	0.0000145	0.00000672	0.00000992	-0.00000791			0.00000838	0.00000734			
	(0.0000102)	(0.0000141)	(0.0000105)	(0.0000144)			(0.0000105)	(0.0000116)			
Precip Sq.	-4.75e-09	-2.69e-09	-3.23e-09	4.75e-10			-2.08e-09	-2.62e-09			
	(2.50e-09)	(3.52e-09)	(2.55e-09)	(3.57e-09)			(2.41e-09)	(2.63e-09)			
Temp*Poor		0.0165		-0.00752	-0.0111*						
		(0.00932)		(0.0106)	(0.00527)						
Temp Sq.*Poor		-0.000456		0.000161							
		(0.000250)		(0.000275)							
Precip*Poor		0.0000191		0.0000342							
		(0.0000207)		(0.0000212)							
Precip Sq.*Poor		-4.75e-09		-7.42e-09							
		(5.07e-09)		(5.19e-09)							
Change Temp									0.00923*	0.0103*	0.00945*
									(0.00363)	(0.00399)	(0.00368)
Change Temp Sq.									-0.000333*	-0.000382*	-0.000337**
									(0.000147)	(0.000157)	(0.000103)
Change Precip									0.00000334	0.00000384	0.00000340
									(0.0000151)	(0.0000125)	(0.00000877)
Change Precip Sq.									-1.12e-09	-1.16e-09	-1.21e-09
									(4.83e-09)	(1.00e-08)	(2.22e-09)
Temperature Function	Quadratic	Quadratic	Quadratic	Quadratic	Linear	Quadratic	Quadratic	Quadratic	Quadratic	Quadratic	Quadratic
GDP Growth or Levels	Growth	Growth	Growth	Growth	Growth	Growth	Growth	Growth	Levels	Levels	Levels
Time Fixed Effects	Year	Year	Year	Year	Region-Year	Region-Year	Region-Year	Region-Year	Region-Year	Year	Region-Year
Country-Specific Time Trend	Quadratic	Quadratic	Quadratic	Quadratic	None	None	None	None	Quadratic	Quadratic	None
Short or Long Run	Short Run	Short Run	Long Run	Long Run	Short Run	Short Run	Short Run	Long Run			
Obs.	6584	6452	5754	5637	6452	6584	6584	5754	6518	6519	6518
R sq.	0.286	0.291	0.321	0.330	0.278	0.267	0.267	0.295	0.371	0.294	0.266
Adj. R sq.	0.221	0.225	0.247	0.253	0.209	0.211	0.211	0.234	0.285	0.229	0.209

Notes: Standard errors in parentheses. Significance stars correspond to * $p < 0.05$, ** $p < 0.01$, *** $p < 0.001$. All models include country fixed effects. Temperature is measured in C. Precipitation is measured in mm/year. Columns represent models specified as follows. (1) Estimates a pooled growth model with quadratic temperature and precipitation, year fixed effects, and a quadratic country time trend. (2) Estimates a growth model with quadratic temperature and precipitation and lags up to 5 years, year fixed effects, and a quadratic country time trend. (3) Estimates a growth model with quadratic temperature and precipitation for rich and poor countries separately, year fixed effects, and a quadratic country time trend. (4) Estimates a growth model with quadratic temperature and precipitation for rich and poor countries separately lagged up to 5 years, year fixed effects, and a quadratic country time trend. (5) Estimates a growth with linear temperature separately for rich and poor countries, region-year fixed effects, and no country time trend. (6) Estimates a pooled growth model with quadratic temperature, region-year fixed effects, and no country time trend. (7) Estimates a pooled growth model with quadratic temperature and precipitation, region-year fixed effects, and no country time trend. (8) Estimates a pooled growth model with quadratic temperature and precipitation lagged up to 5 years, region-year fixed effects, and no country time trend. (9) Estimates a pooled levels model with quadratic temperature and precipitation, region-year fixed effects, and a quadratic country time trend. (10) Estimates a pooled levels model with quadratic temperature and precipitation, year fixed effects, and a quadratic country time trend. (11) Estimates a pooled levels model with quadratic temperature and precipitation, region-year fixed effects, and no country time trend.

Supplementary Table 2 | GeoMIP G1 Experiment Models. Models used to construct mean ensemble for climate variable changes from solar geoengineering.

**Temperature**	**Precipitation**
BNU-ESM	BNU-ESM
CanESM2	CanESM2
CCSM4	CCSM4
CESM-CAM5.1-FV2	CESM-CAM5.1-FV2
EC-EARTH	EC-EARTH
HadCM3	HadCM3
HadGEM2-ES	HadGEM2-ES
IPSL-CM5A-LR	IPSL-CM5A-LR
MIROC-ESM	MIROC-ESM
MPI-ESM-LR	MPI-ESM-LR
NorESM1-M	NorESM1-M
CSIRO-Mk3L-1.2	CSIRO-Mk3L-1.2

Supplementary Table 3 | Percentage of countries with an absolute loss in 2099 compared to 2010. Values represent median projections for SSP3. Columns represent models specified as follows. (1) Estimates a pooled growth model with quadratic temperature and precipitation, year fixed effects, and a quadratic country time trend. (2) Estimates a growth model with quadratic temperature and precipitation and lags up to 5 years, year fixed effects, and a quadratic country time trend. (3) Estimates a growth model with quadratic temperature and precipitation for rich and poor countries separately, year fixed effects, and a quadratic country time trend. (4) Estimates a growth model with quadratic temperature and precipitation for rich and poor countries separately lagged up to 5 years, year fixed effects, and a quadratic country time trend. (5) Estimates a growth with linear temperature separately for rich and poor countries, region-year fixed effects, and no country time trend. (6) Estimates a pooled growth model with quadratic temperature, region-year fixed effects, and no country time trend. (7) Estimates a pooled growth model with quadratic temperature and precipitation, region-year fixed effects, and no country time trend. (8) Estimates a pooled growth model with quadratic temperature and precipitation lagged up to 5 years, region-year fixed effects, and no country time trend. (9) Estimates a pooled levels model with quadratic temperature and precipitation, region-year fixed effects, and a quadratic country time trend. (10) Estimates a pooled levels model with quadratic temperature and precipitation, year fixed effects, and a quadratic country time trend. (11) Estimates a pooled levels model with quadratic temperature and precipitation, region-year fixed effects, and no country time trend.

Scenario	(1)	(2)	(3)	(4)	(5)	(6)	(7)	(8)	(9)	(10)	(11)
RCP8.5 (2099)	43.03	46.67	36.97	21.21	16.36	10.91	10.3	6.06	0	0	0
Geo Stabilized RCP8.5 (2099)	0	0	0	0	0	0	0	0	0	0	0
Geo Mirrored RCP8.5 (2099)	10.91	0	8.48	0	28.48	18.79	20	5.45	0	0	0
No Climate Change (2099)	0	0	0	0	0	0	0	0	0	0	0

Supplementary Table 4 | Percentage of countries with a relative loss compared with no climate change in 2099. Values represent median projections for SSP3. Columns as in Table S3.

Scenario	(1)	(2)	(3)	(4)	(5)	(6)	(7)	(8)	(9)	(10)	(11)
RCP8.5 (2099)	76.36	100	72.73	100	35.76	64.24	63.64	66.06	76.97	76.97	75.76
Geo Stabilized RCP8.5 (2099)	32.73	53.33	32.12	57.58	39.39	24.24	29.7	24.24	32.73	32.73	32.12
Geo Mirrored RCP8.5 (2099)	32.12	0	35.76	0	92.73	41.82	41.82	39.39	36.36	36.36	36.36
No Climate Change (2099)	0	0	0	0	0	0	0	0	0	0	0

Supplementary Table 5 | Projected country-level Gini Coefficients.. Values represent median projections for SSP3. Columns as in Table S3.

Scenario	(1)	(2)	(3)	(4)	(5)	(6)	(7)	(8)	(9)	(10)	(11)
RCP8.5 (2099)	0.8740	0.6452	0.8572	0.7626	0.8335	0.8382	0.8372	0.7544	0.5875	0.5882	0.5876
Geo Stabilized RCP8.5 (2099)	0.5735	0.5703	0.5736	0.5727	0.5834	0.5849	0.5840	0.5842	0.5823	0.5823	0.5823
Geo Mirrored RCP8.5 (2099)	0.4595	0.5063	0.4379	0.5621	0.3488	0.4499	0.4538	0.4568	0.5772	0.5764	0.5771
No Climate Change (2099)	0.5824	0.5824	0.5824	0.5824	0.5824	0.5824	0.5824	0.5824	0.5824	0.5824	0.5824
(2010)	0.7482	0.7482	0.7482	0.7482	0.7482	0.7482	0.7482	0.7482	0.7482	0.7482	0.7482
